# Green Synthesis of Indeno[1,2-b]quinoxalines Using β-Cyclodextrin as Catalyst

**DOI:** 10.3390/molecules27020580

**Published:** 2022-01-17

**Authors:** Li-Guo Liao, Meng-Meng Song, Jun-Feng Feng, Min Tan, Fan Liu, Zhen-Jiang Qiu, Sheng Zhang, Bang-Jing Li

**Affiliations:** 1 Key Laboratory of Mountain Ecological Restoration and Bioresource Utilization, Chengdu Institute of Biology, Chinese Academy of Sciences, Chengdu 610041, China; liaolg@cib.ac.cn (L.-G.L.); tanmin@cib.ac.cn (M.T.); liufan@cib.ac.cn (F.L.); qzj19890323qzj@126.com (Z.-J.Q.); 2 University of Chinese Academy of Sciences, Beijing 100049, China; 3 State Key Laboratory of Polymer Materials Engineering, Polymer Research Institute of Sichuan University, Chengdu 610065, China; songmengmeng@stu.scu.edu.cn (M.-M.S.); fengjf408@stu.scu.edu.cn (J.-F.F.)

**Keywords:** indeno[1,2-b]quinoxalines, *β*-CD, environmentally friendly method, reusable catalyst

## Abstract

An efficient, mild, and green method was developed for the synthesis of indeno[1,2-b]quinoxaline derivatives via o-phenylenediamine (OPD) and 2-indanone derivatives utilizing *β*-cyclodextrin (*β*-CD) as the supramolecular catalyst. The reaction can be carried out in water and in a solid state at room temperature. *β*-CD can also catalyze the reaction of indan-1,2-dione with OPD with a high degree of efficiency. Compared to the reported methods, this procedure is milder, simpler, and less toxic, making it an eco-friendly alternative. In addition, the *β*-CD can be recovered and reused without the loss of activity.

## 1. Introduction

**Figure 1:**
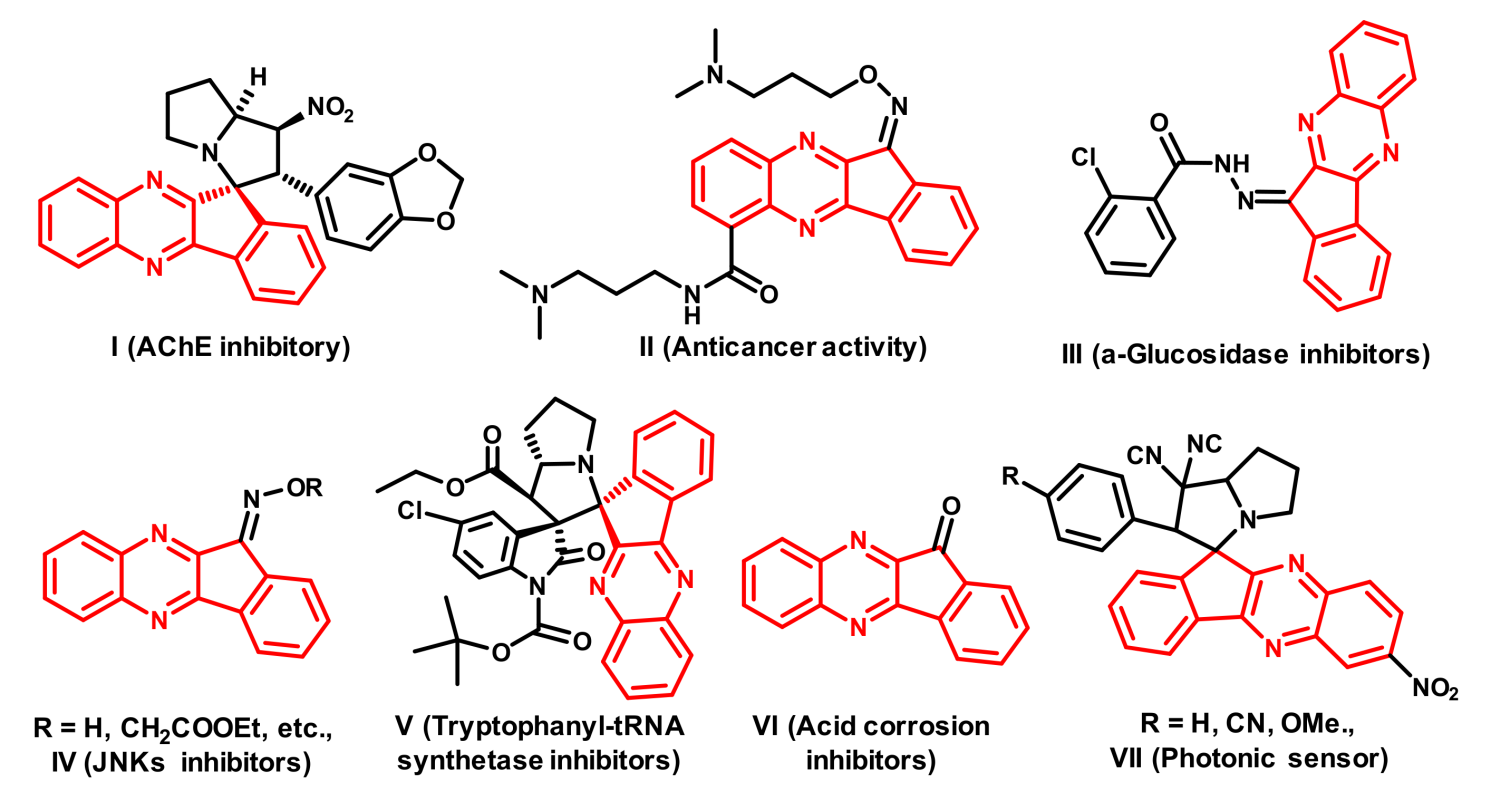
Some active compounds of the indeno[1,2-b]quinoxaline skeleton.

Indeno[1,2-b]quinoxaline skeleton compounds exist in a wide number of drug candidates [1,2,3,4,5,6,7]. They exhibit diverse biological properties, such as acetylcholinesterase (AChE) inhibitory activity (**I**) [1], antitumor activity (**II**) [2,3], α-glucosidase inhibition (**III**) [4], c-Jun N-terminal kinase (JNK) inhibition (**IV**) [5,6], and tryptophan-tRNA synthase (TrpRS) inhibition (**V**) [7]. Furthermore, functionally substituted indeno[2,3-b]quinoxalines show potential as acid corrosion inhibitors for mild steel surfaces (**VI**) [8] and can be used as a photonic sensor to detect fluorescent dyes in the waste effluents of textiles, paper, dyes, and other industrial products (**VII**) [9] (Figure 1). 

**Scheme 1:**
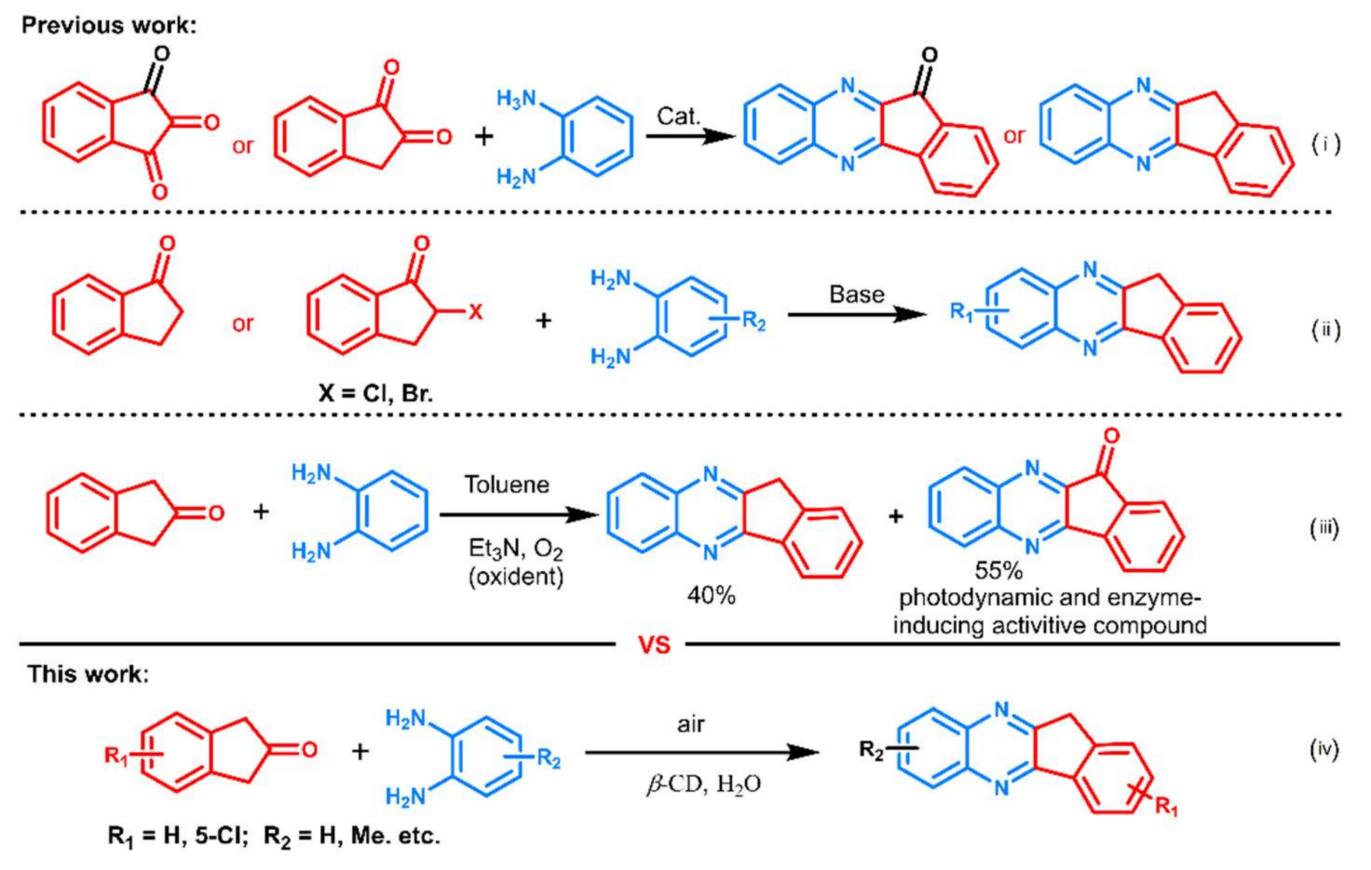
Research background and our initial proposal.

Therefore, a great deal of attention has been paid to the construction of indeno[1,2-b]quinoxaline skeleton compounds [10]. Among these methods, the condensation of OPD with indan-1,2-diones or 1,2,3-indanetriones [11,12,13] (Scheme 1, Equation (i)) and the α-halogenation of 1-indanones with OPDs are the most commonly practiced [14,15] (Scheme 1, Equation (ii)). In addition to these strategies, other oxidation methods or ways of using other types of substrates have also been developed, such as the reaction between 2-indanone and OPD [16] (Scheme 1, Equation (iii)). However, most of these methodologies still suffer from some disadvantages, such as the use of toxic or strongly alkaline catalysts, hazardous solvents, a complicated process, or the formation of side products. In recent decades, the increasing awareness of environmental security and global warming has attracted worldwide attention to the use of eco-friendly and atom-economical conditions in organic synthesis [17,18]. Therefore, it is of great use to develop a simple, mild, and environmentally benign methodology for the synthesis of indeno[1,2-b]quinoxaline derivatives.

Cyclodextrins (CDs) are cyclic oligosaccharides made from starch and generally consist of six, seven, or eight D-(+)-glucose units referred to as *α*-, *β*-, and *γ*-CD, respectively. They possess a truncated cone structure with a hydrophobic cavity and a hydrophilic surface [19]. This unique structure renders CDs with aqueous solubility and the ability to form host–guest inclusion complexes with guest molecules. The selective inclusion process is similar to the behavior of the enzymatic catalytic reaction [20]. Therefore, CDs have attracted much attention as a biomimetic catalyst and have been demonstrated to promote a broad range of chemical reactions in benign, mild conditions [21,22]. For example, Nageswar et al. reported the synthesis of quinoxaline derivatives from OPD and phenacyl bromides in the presence of *β*-CD in water [23], and Kakulapati et al. reported one-pot synthesis of quinoxalines by OPD with aerobic oxidation of benzoins using Ru/C in Ra-Me-*β*-CD [24]. However, these two methods still require heating or a Ru/C catalyst to assist with the reaction. In this study, we explored the synthesis of indeno[1,2-b]quinoxalines at room temperature using *β*-CD as a catalyst (Scheme 1, Equation (iv)). The results showed that *β*-CD exhibits high catalytic performance for the construction of indeno[1,2-b]quinoxaline skeleton compounds from 2-indanone and OPD under mild conditions in an aqueous medium. In addition, a plausible mechanism was proposed on the basis of two-dimensional nuclear magnetic resonance spectroscopy (2D NMR).

## 2. Results and Discussion

First, using 2-indanone and OPD as template substrates, we compared the catalytic effects of the base and of different CDs at room temperature (Table 1, entries 2–8) and ascertained whether the reaction could be carried out in the absence of a catalyst (Table 1, entry 1). We found there were trace products in the water without a catalyst, and that the CDs showed better catalytic activity than the base catalyst, triethylamine (Table 1, entry 8). Among the different CDs, *β*-CD gave **2.3aa** in the best yield (Table 1, entries 2–4), which suggested that the size of the cavity of the CDs (*α*-CD: 5.7 Å cavity, *β*-CD: 7.8 Å cavity, and *γ*-CD: 9.5 Å cavity) plays an important role in the catalysis [19] and that *β*-CD had the optimal cavity size. The catalytic efficiencies of different *β*-CDs were varied (Table 1, entries 3, 5–7). We found that the substitution markedly decreased the catalytic efficiency of *β*-CD. When three hydroxyl groups of *β*-CD were replaced by methyl, the yield decreased from 85% to 38%, suggesting that the hydroxyl groups of *β*-CD were beneficial to the catalysis.

We also carried out a solvent-free reaction (Table 1, entry 9). We found that there was a 70% yield by grinding substrates for 0.5 h. However, the yield of this solid-state reaction did not improve with increased time, which might indicate insufficient contact between the substrates and the catalyst. One-pot solid-state synthesis is an excellent green technique. Although the yield of indeno[1,2-b]quinoxaline is only moderate, this *β*-CD-catalyzed solid-state synthesis method is still a potential alterative for the construction of indeno[1,2-b]quinoxaline skeleton compounds. We also carried out the reaction in a nitrogen atmosphere. We found that only trace amounts of the product were produced (Table 1, entry 10), indicating that oxygen is necessary for the reaction.

Choosing *β*-CD as the catalyst, we screened the reaction solvents: dimethyl sulfoxide (DMSO), *N*,*N*-dimethyl formamide (DMF), and ethanol (EtOH) (Table 1, entries 11–13). We found that water gave the best result.

The influence of the reaction time, catalyst dosage, temperature, and substrate dosage on the reaction was also investigated (Table 1, entries 13–17). It can be seen that high temperatures were unfavorable to the reaction. This finding indicated that the inclusion complex between substrates and *β*-CD is very important for the catalysis, since high temperatures reduce the inclusion complexation. Finally, we found the optimal reaction conditions are as follows: molar ration of **2.2a**/**2.1a** is 1.2: 1, reaction time of 12 h, and concentration of *β*-CD of 15 mol% (Table 1, entry 16).

**Figure 2:**
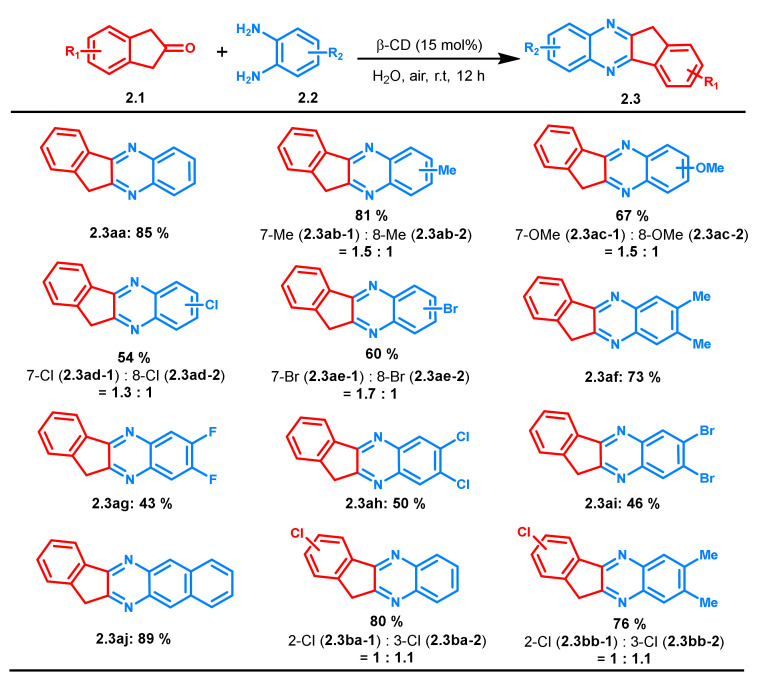
Scope of the various substituted 2-indanones (**2.1**) and o-phenylenediamines (**2.2**) to synthesis of indeno[1,2-b]quinoxalines (**2.3**) in optimal conditions.

After determining the optimal conditions, we extended the process to other substrates. The results are shown in Figure 2. We observed that the substitution groups on OPD decreased the product yield significantly, while the substituents on 2-indanone had relatively little effect on the yield. For example, the product (**2.3ad-1** and **2.3ad-2**) yield was only 54% when the OPD was substituted in 4-Cl. However, the product (**2.3ba-1** and **2.3ba-2**) yield was 80% when the chlorine substitution occurred on the benzene ring of 2-indanone. The substitutions of the electron donor groups and of the electron withdrawing groups on the OPD both led to a decrease in product yield. A double substitution caused a greater reduction in the yield than a single substitution. It is interesting that 2,3-diaminonaphthalene reacted with 2-indanone better than the OPD. The yield of the corresponding product (**2.3aj**) was 89%. It is known that the inclusion constant of naphthalene and *β*-CD is greater than that of benzene and its derivatives [25,26,27]. Therefore, it is supposed that the complexation of *β*-CD and OPD and its derivatives played a key role in the catalysis.

**Figure 3:**
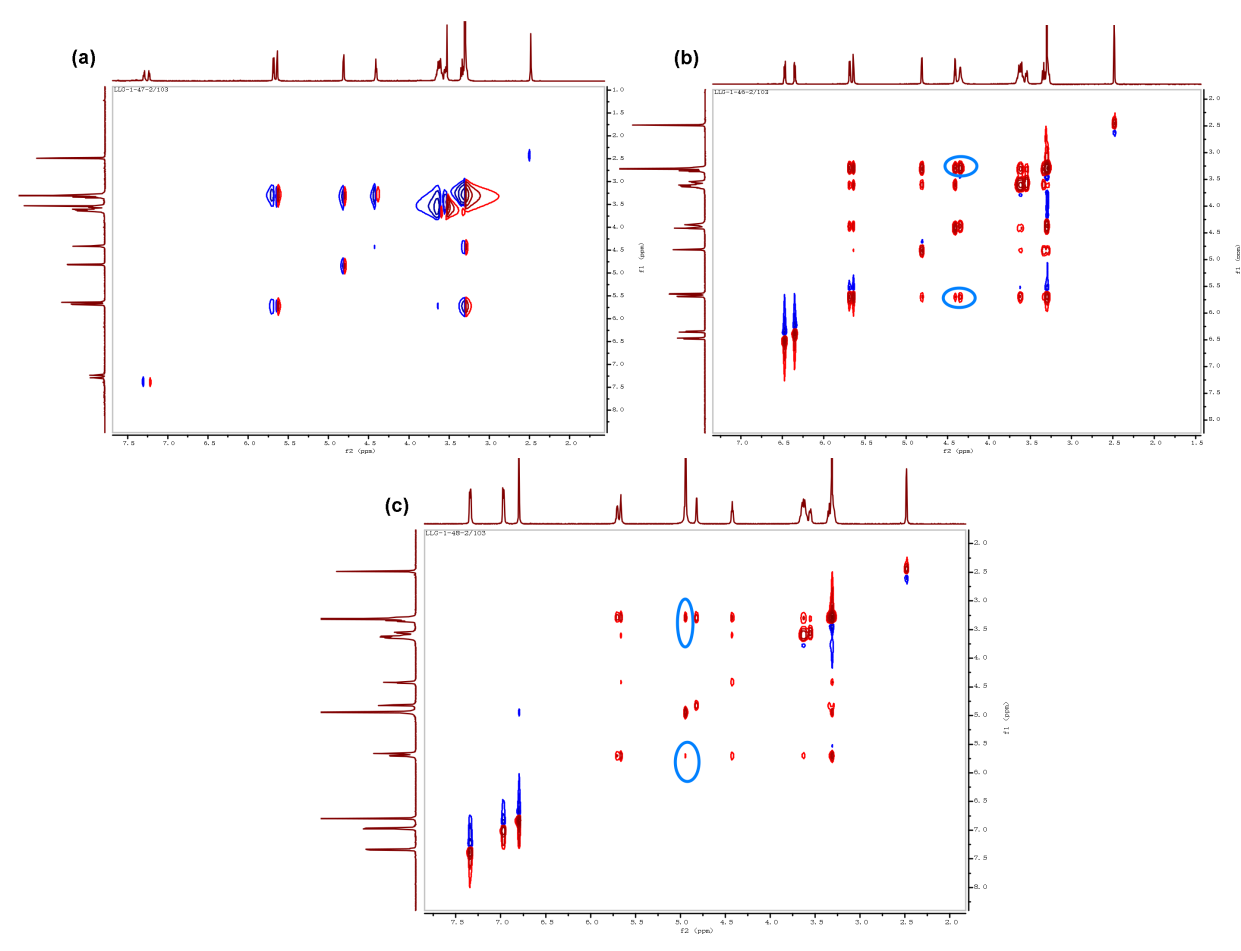
The 2D NOESY NMR spectra of freeze-dried: (**a**) 2-indanone (1.0 eqv.) and *β*-CD (15 mol%), (**b**) o-phenylenediamine (1.2 eqv.) and *β*-CD (15 mol%), and (**c**) 2,3-diaminonaphthalene (1.2 eqv.) and *β*-CD (15 mol%). Mixed after stirring in water for 12 h.

In order to investigate the mechanism, the complexations of *β*-CD mixed with three substrates (2-indanone, OPD, and 2,3-diaminonaphthalene) were investigated by 2D NOESY NMR. As shown in Figure 3a, the characteristic peaks of 2-indanone, δ 7.29 and 7.24 (low-field field) and δ 3.53 (the high field), were not related with the peaks of *β*-CD, indicating that 2-indanone cannot enter the *β*-CD cavity. On the contrary, the amino characteristic peak of **2.2a** at δ 4.35 and the amino characteristic peak of **2.2j** at δ 4.94 showed a clear relation with the peak of *β*-CD (C_2_-OH and C_3_-OH) [28] (Figure 3b,c, the blue line). In addition, **2.2a** and **2.2j** were also related with *β*-CD near δ 3.30 (see the detailed information in Figures S53 and S54). This suggests that OPD and 2,3-diaminonaphthalene can be included in the *β*-CD cavity to form the inclusion complex.

**Scheme 2:**
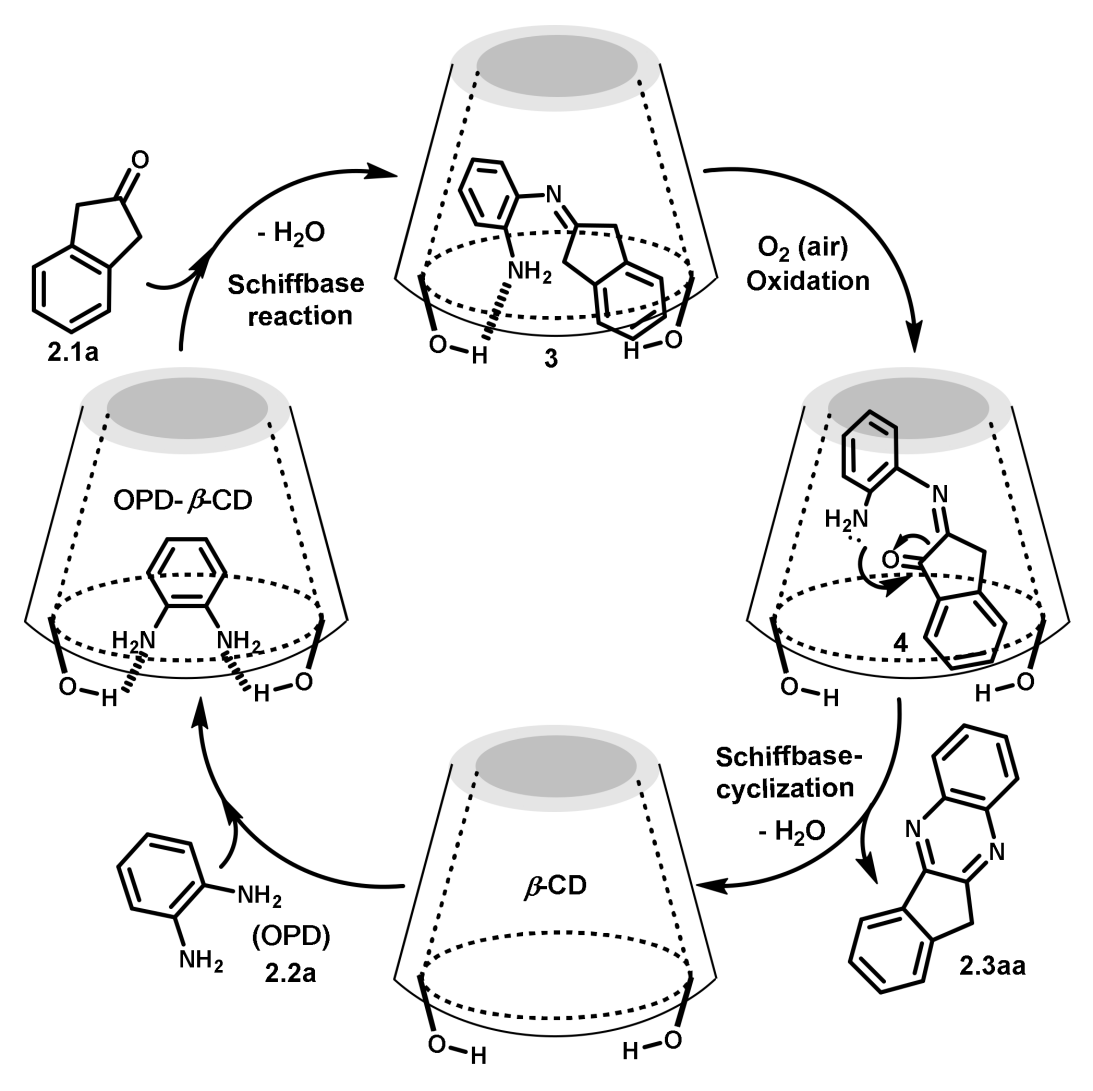
A plausible mechanism for the reaction.

Based on the above data and the reports documented in the literature [29,30,31,32], a plausible reaction mechanism is proposed in Scheme 2. First, OPD is included in the cavity of *β*-CD to form the OPD-*β*-CD inclusion complex. The reactivity of the amino groups of OPD increases due to the formation of intermolecular hydrogen bonding with the hydroxy groups in *β*-CD. Then, the first Schiff base reaction takes place, removing a mole of water to form intermediate **3** [31]. The generated intermediate **3** is immediately oxidized by oxygen in the air to form the intermediate **4**, leading to the second Schiff base reaction [32]. The presence of intermediates **3** and **4** was confirmed by high-resolution mass spectrometry (HRMS). We found that there were clear intermediate peaks for **3** and **4** in the HRMS spectrum of the reaction solution after a one-hour reaction (Figure S55). Previous studies reported that a base was needed for the monocarbonylated compounds oxidation during quinoxaline synthesis [30]. It is interesting that a high yield was still obtained by using *β*-CD as a catalyst in the absence of a base in this study. Ji et al. reported that *β*-CD could improve the synthesis of 2-phenylbezimidazole using air as an oxidant, since the oxidative cyclodehydrogenation of the Schiff base intermediates was promoted inside the *β*-CD cavity [29]. Therefore, it is supposed that the mechanism of *β*-CD catalysis in this study is also due to the promotion of oxidative cyclodehydrogenation. Finally, the *β*-CD catalyst returns to its initial state by releasing the product from its cavity.

**Figure 4:**
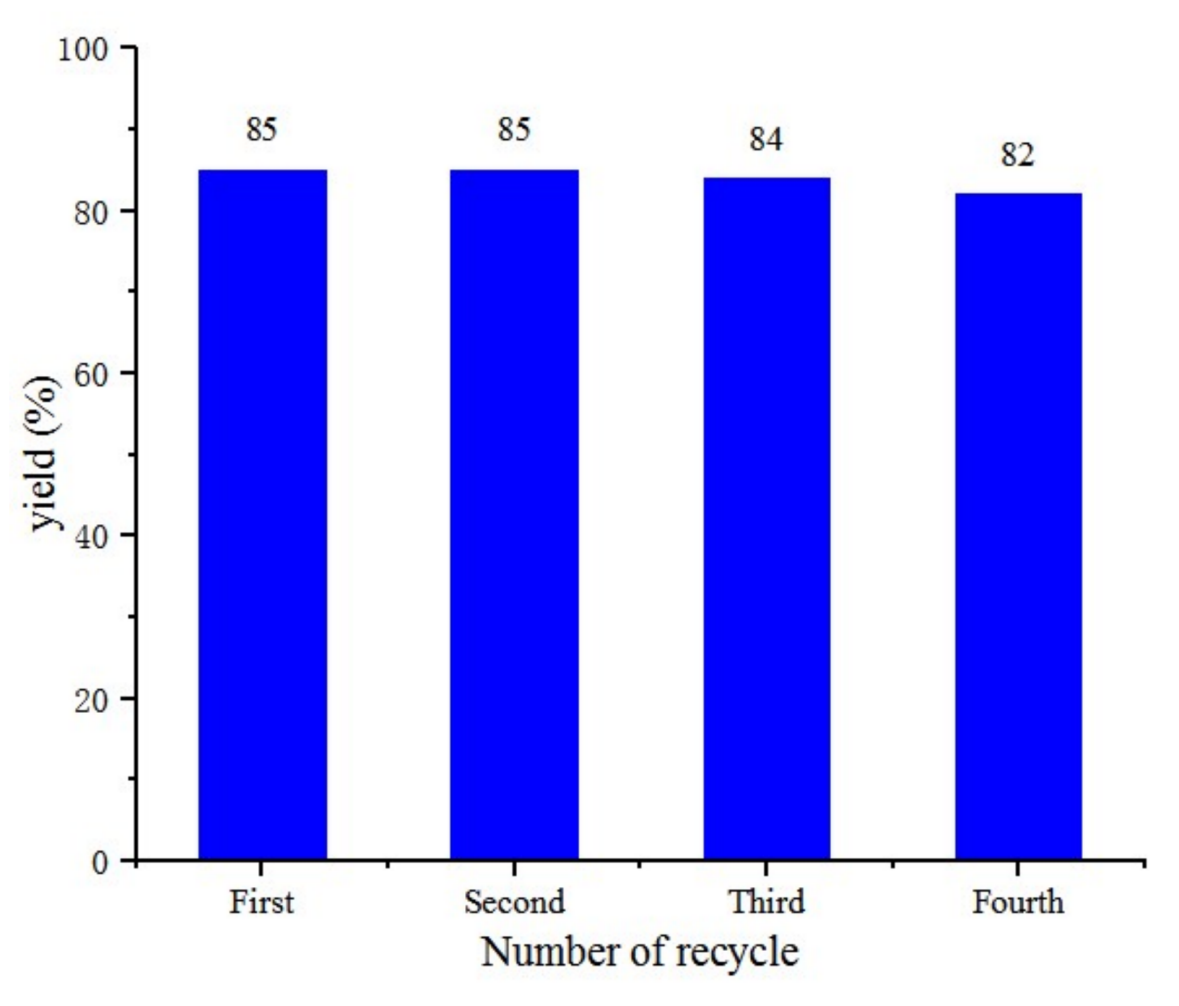
Recyclability of the catalyst.

After the completion of the reaction, the *β*-CD can be recycled after the extraction of the products by using ethyl acetate. The reusability of the catalyst was examined four times for the synthesis of **2.3aa,** and there was no significant reduction in the yield (Figure 4).

**Scheme 3:**
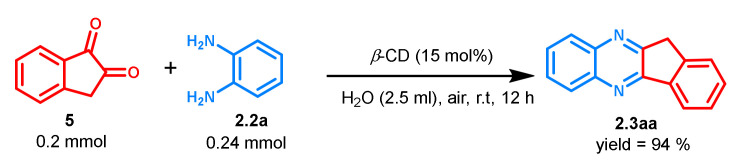
The application of cyclodextrin biomimetic catalysis.

*β*-CD can also catalyze the reaction of indan-1,2-dione with OPD (Scheme 3). Previous research on the reaction of indan-1,2-dione with OPD used ammonium bifluoride as a catalyst and methanol–water as a solvent [11]. The strategy developed in this study is not only more environmentally friendly, but also more efficient.

## 3. Materials and Methods

### 3.1. General

Commercial reagents were purchased from Sigma-Aldrich, Aladdin Reagent Database Inc. and Bide pharmatech Ltd. (without further purification). ^1^H, ^13^C NMR, and NOESY spectra were recorded on a Bruker AVANCE 600 instrument using DMSO-d_6_ or CDCl_3_ as a solvent. Chemical shifts δ are expressed in parts per million (ppm) and internally referenced to tetramethylsilane (TMS). The coupling constant *J* is reported in Hz. The following abbreviations are used: s = singlet, d = doublet, t = triplet, q = quartet, and m = multiplet. High-resolution mass spectra (HRMS) were obtained on a micrOTOF-QII mass spectrometer (Bruker, Germany) or a Vion^®^ IMS QTof mass spectrometer (Waters, UK). The ionization method chosen was electrospray ionization (ESI) operated in positive ion mode. All the reaction procedures were monitored by TLC using 40 precoated sheets of silica gel G/UV-254 of 0.25 mm thickness, Merck 60 F254 (BioLong, China). TLC plates were visualized by exposure to ultraviolet light and/or by exposure to iodine vapors, and the product was obtained by chromatography performed on silica gel (200–300 mesh).

### 3.2. General Procedure for the Synthesis of Compounds **2.3**(**aa-bb**)

2-indanone (0.1 mmol, 26.4 mg), OPD (0.1 mmol, 26.0 mg), and *β*-CD (15 mol%, 34.1 mg) were mixed in distilled water (2.5 mL). The reaction mixture was stirred at room temperature (25.0 °C) for 12 h. After the completion of the reaction, the reaction mixture was extracted with ethyl acetate. Then, the organic layer was dried through Na_2_SO_4_ and concentrated in vacuo. Finally, the crude product was purified by flash chromatography on a short silica gel (eluent: petroleum ether/ethyl acetate =10:1) to afford 37.1 mg (85%) of **2.3aa**. Other product compounds **2.3(ab**-**bb)** were synthesized by similar methods.

11*H*-indeno[1,2-b]quinoxaline (**2.3aa**)

Yellow solid; 85% yield; ^1^H NMR (600 MHz, CDCl_3_) δ: 8.34–8.26 (m, 1H), 8.21 (dd, *J* = 8.0, 1.7 Hz, 1H), 8.11 (dd, *J* = 8.0, 1.7 Hz, 1H), 7.75 (dddd, *J* = 18.0, 8.3, 6.9, 1.6 Hz, 2H), 7.68 (dt, *J* = 7.3, 1.2 Hz, 1H), 7.60–7.52 (m, 2H), 4.17 (s, 2H); ^13^C NMR (100 MHz, CDCl_3_) δ: 159.48, 154.67, 143.52, 142.07, 141.26, 138.04, 131.15, 129.26, 129.21, 128.97, 128.84, 128.06, 125.83, 122.70, 35.98; HRMS (ESI) calcd for C_15_H_11_N_2_^+^ (M+H)^+^ 219.0917, found 219.0912.

7/8-methyl-11*H*-indeno[1,2-b]quinoxaline (**2.3ab-1** and **2.3ab-2**)

**2.3ab-1** and **2.3ab-2** cannot be separated through column chromatography; yellow solid; 81% yield; ^1^H NMR (600 MHz, CDCl_3_) δ: 8.23–8.15 (m, 1H), 7.97 (dd, *J* = 46.4, 8.4 Hz, 1H), 7.85 (dd, J = 51.9, 1.8 Hz, 1H), 7.59 (dddd, *J* = 5.0, 3.2, 2.0, 1.0 Hz, 1H), 7.55–7.44 (m, 3H), 4.06 (s, 2H), 2.57 (s, 3H); ^13^C NMR (150 MHz, CDCl_3_) δ 159.29, 158.43, 154.41, 153.76, 143.44, 143.21, 142.02, 141.26, 140.35, 139.63, 139.53, 139.19, 138.17, 131.29, 130.89, 130.84, 130.71, 128.67, 128.40, 128.23, 128.00, 127.89, 125.68, 122.53, 122.39, 77.25, 77.04, 76.83, 35.93, 35.84, 21.73, 21.70; HRMS (ESI) calcd for C_16_H_13_N_2_^+^ (M + H)^+^ 233.1073, found 233.1068.

7-methoxy-11*H*-indeno[1,2-b]quinoxaline (**2.3ac-1**)

White solid; 41% yield; ^1^H NMR (600 MHz, CDCl_3_) δ: 8.27 (d, *J* = 6.9 Hz, 1H), 7.99 (d, *J* = 9.1 Hz, 1H), 7.67 (d, *J* = 6.8 Hz, 1H), 7.58–7.50 (m, 3H), 7.38 (dd, *J* = 9.1, 2.8 Hz, 1H), 4.13 (s, 2H), 4.00 (s, 3H); ^13^C NMR (150 MHz, CDCl_3_) δ: 160.41, 156.90, 143.69, 138.13, 137.25, 130.94, 129.80, 127.99, 125.81, 122.54, 121.59, 107.13, 77.20, 76.99, 76.78, 55.81, 35.81; HRMS (ESI) calcd for C_16_H_13_N_2_O^+^ (M + H)^+^ 249.1022, found 249.1023.

8-methoxy-11*H*-indeno[1,2-b]quinoxaline (**2.3ac-2**)

White solid; 26% yield; ^1^H NMR (600 MHz, CDCl_3_) δ: 8.22 (dd, *J* = 5.3, 3.5 Hz, 1H), 8.07 (d, *J* = 9.1 Hz, 1H), 7.69–7.61 (m, 1H), 7.53 (dd, *J* = 5.6, 3.1 Hz, 2H), 7.44 (d, *J* = 2.8 Hz, 1H), 7.41 (dd, *J* = 9.1, 2.8 Hz, 1H), 4.13 (s, 2H), 3.99 (s, 3H); ^13^C NMR (150 MHz, CDCl_3_) δ: 160.10, 159.58, 152.53, 142.78, 142.74, 138.39, 137.85, 130.44, 130.08, 127.95, 125.70, 122.12, 121.82, 107.24, 77.21, 76.99, 76.78, 55.74, 36.00; HRMS (ESI) calcd for C_16_H_13_N_2_O^+^ (M + H)^+^ 249.1022, found 249.1027.

7-chloro-11*H*-indeno[1,2-b]quinoxaline (**2.3ad-1**)

Yellow solid; 31% yield; ^1^H NMR (600 MHz, CDCl_3_) δ 8.24 (d, J = 7.6 Hz, 1H), 8.15 (d, J = 2.3 Hz, 1H), 8.01 (d, J = 8.8 Hz, 1H), 7.65 (td, J = 7.1, 6.7, 3.1 Hz, 2H), 7.59–7.50 (m, 2H), 4.12 (s, 2H); ^13^C NMR (150 MHz, CDCl_3_) δ 159.70, 155.26, 143.74, 142.28, 139.71, 137.54, 134.97, 131.61, 130.06, 129.63, 128.19, 128.08, 125.84, 123.03, 35.91; HRMS (ESI) calcd for C_15_H_10_ClN_2_^+^ (M + H)^+^ 253.0527, found 253.0517.

8-chloro-11*H*-indeno[1,2-b]quinoxaline (**2.3ad-2**)

White solid; 23% yield; ^1^H NMR (600 MHz, CDCl_3_) δ 8.22–8.16 (m, 1H), 8.09–8.00 (m, 2H), 7.69–7.60 (m, 2H), 7.58–7.46 (m, 2H), 4.10 (s, 2H); ^13^C NMR (150 MHz, CDCl3) δ 160.43, 154.81, 143.45, 141.50, 140.56, 137.67, 134.32, 131.37, 130.28, 130.07, 128.13, 128.00, 125.81, 122.72, 35.93; HRMS (ESI) calcd for C_15_H_10_ClN_2_^+^ (M + H)^+^ 253.0527, found 253.0520.

7-bromo-11*H*-indeno[1,2-b]quinoxaline (**2.3ae-1**)

Yellow solid; 38% yield; ^1^H NMR (600 MHz, CDCl_3_) δ: 8.33 (d, *J* = 2.1 Hz, 1H), 8.22 (d, *J* = 7.4 Hz, 1H), 7.94 (d, J = 8.8 Hz, 1H), 7.77 (dd, *J* = 8.8, 2.2 Hz, 1H), 7.65 (d, *J* = 7.4 Hz, 1H), 7.58–7.52 (m, 2H), 4.11 (s, 2H); ^13^C NMR (150 MHz, CDCl_3_) δ: 159.81, 155.32, 143.70, 142.78, 140.00, 137.65, 132.14, 131.53, 130.21, 128.17, 125.82, 122.97, 122.92, 35.94; HRMS (ESI) calcd for C_15_H_10_BrN_2_^+^ (M + H)^+^ 297.0022, found 297.0027.

8-bromo-11*H*-indeno[1,2-b]quinoxaline (**2.3ae-2**)

Yellow solid; 22% yield; ^1^H NMR (600 MHz, CDCl_3_) δ: 8.30–8.20 (m, 2H), 8.01 (d, *J* = 8.8 Hz, 1H), 7.81 (dd, *J* = 8.9, 2.2 Hz, 1H), 7.70–7.63 (m, 1H), 7.60–7.49 (m, 2H), 4.13 (s, 2H); ^13^C NMR (150 MHz, CDCl_3_) δ: 160.40, 154.93, 143.54, 141.85, 140.88, 137.71, 132.66, 131.42, 131.36, 130.44, 128.16, 125.84, 122.78, 122.41, 35.96; HRMS (ESI) calcd for C_15_H_10_BrN_2_^+^ (M + H)^+^ 297.0022, found 297.0024.

7,8-dimethyl-11*H*-indeno[1,2-b]quinoxaline (**2.3af**)

Pale yellow solid; 73% yield; ^1^H NMR (600 MHz, CDCl_3_) δ: 8.31–8.15 (m, 1H), 7.92 (s, 1H), 7.83 (s, 1H), 7.64 (ddd, *J* = 5.4, 2.7, 1.0 Hz, 1H), 7.55–7.48 (m, 2H), 4.11 (s, 2H), 2.50 (t, *J* = 1.2 Hz, 6H); ^13^C NMR (150 MHz, CDCl_3_) δ: 158.46, 153.73, 143.26, 140.82, 140.15, 139.47, 139.16, 138.40, 130.62, 128.44, 128.21, 127.90, 125.71, 122.36, 35.94, 20.29, 20.25; HRMS (ESI) calcd for C_17_H_15_N_2_^+^ (M + H)^+^ 247.1230, found 247.1229.

7,8-difluoro-11*H*-indeno[1,2-b]quinoxaline (**2.3ag**)

Yellow solid; 43% yield; ^1^H NMR (600 MHz, CDCl_3_) δ: 8.22 (d, *J* = 7.3 Hz, 1H), 7.91 (dd, *J* = 10.7, 8.2 Hz, 1H), 7.84 (dd, *J* = 10.6, 8.1 Hz, 1H), 7.70–7.65 (m, 1H), 7.60–7.53 (m, 2H), 4.14 (s, 2H); ^13^C NMR (150 MHz, CDCl_3_) δ: 159.81, 154.93, 143.35, 139.29, 138.30, 138.23, 137.62, 131.47, 128.19, 125.84, 122.75, 115.04, 114.94, 114.83, 35.88; HRMS (ESI) calcd for C_15_H_9_F_2_N_2_^+^ (M + H)^+^ 255.0728, found 255.0735.

7,8-dichloro-11*H*-indeno[1,2-b]quinoxaline (**2.3ah**)

Yellow solid; 50% yield; ^1^H NMR (600 MHz, CDCl_3_) δ: 8.29 (s, 1H), 8.24 (d, *J* = 7.5 Hz, 1H), 8.21 (s, 1H), 7.68 (d, *J* = 7.4 Hz, 1H), 7.62–7.53 (m, 2H), 4.15 (s, 2H); ^13^C NMR (150 MHz, CDCl_3_) δ: 160.70, 155.61, 143.68, 140.95, 139.99, 137.45, 133.58, 132.96, 131.78, 129.82, 129.66, 128.27, 125.88, 123.00, 77.20, 76.99, 76.78, 35.95; HRMS (ESI) calcd for C_15_H_9_Cl_2_N_2_^+^ (M + H)^+^ 287.0137, found 287.0145.7,8-dibromo-11*H*-indeno[1,2-b]quinoxaline (**2.3ai**).

Yellow solid; 46% yield; ^1^H NMR (600 MHz, CDCl_3_) δ: 8.48 (s, 1H), 8.40 (s, 1H), 8.23 (d, *J* = 7.5 Hz, 1H), 7.68 (d, *J* = 7.5 Hz, 1H), 7.62–7.53 (m, 2H), 4.14 (s, 2H); ^13^C NMR (150 MHz, CDCl_3_) δ: 160.80, 155.66, 143.76, 141.47, 140.51, 137.44, 133.23, 133.05, 131.83, 128.29, 125.89, 125.54, 124.87, 123.04, 35.98; HRMS (ESI) calcd for C_15_H_9_Br_2_N_2_^+^ (M + H)^+^ 374.9127, found 374.9125.

13*H*-benzo[g]indeno[1,2-b]quinoxaline (**2.3aj**)

Yellow solid; 89% yield; ^1^H NMR (600 MHz, CDCl_3_) δ: 8.71 (s, 1H), 8.62 (s, 1H), 8.30 (dd, *J* = 7.3, 1.5 Hz, 1H), 8.13–8.08 (m, 2H), 7.66 (dt, *J* = 7.6, 1.1 Hz, 1H), 7.60–7.53 (m, 4H), 4.18 (s, 2H); ^13^C NMR (150 MHz, CDCl_3_) δ 160.36, 155.22, 143.93, 138.82, 138.16, 137.88, 133.52, 133.20, 131.72, 128.34, 128.30, 128.17, 127.35, 127.08, 126.50, 126.41, 125.91, 123.06, 35.95; HRMS (ESI) calcd for C_19_H_13_N_2_^+^ (M + H)^+^ 269.1073, found 269.1067.

2-chloro-11*H*-indeno[1,2-b]quinoxaline (**2.3ba-1**)

White solid; 42% yield; ^1^H NMR (600 MHz, CDCl_3_) δ: 8.21–8.13 (m, 2H), 8.10 (dd, *J* = 8.0, 1.8 Hz, 1H), 7.80–7.70 (m, 2H), 7.66 (d, *J* = 1.8 Hz, 1H), 7.52 (dd, *J* = 8.2, 1.8 Hz, 1H), 4.14 (s, 2H); ^13^C NMR (150 MHz, CDCl_3_) δ: 158.89, 153.60, 144.93, 142.07, 141.27, 137.16, 136.61, 129.46, 129.21, 129.08, 129.01, 128.67, 126.17, 123.71, 77.21, 77.00, 76.79, 35.80; HRMS (ESI) calcd for C_15_H_10_ClN_2_^+^ (M + H)^+^ 253.0527, found 253.0517.

3-chloro-11*H*-indeno[1,2-b]quinoxaline (**2.3ba-2**)

White solid; 38% yield; ^1^H NMR (600 MHz, CDCl_3_) δ: 8.22 (d, *J* = 2.0 Hz, 1H), 8.17–8.15 (m, 1H), 8.09 (dd, *J* = 7.9, 1.8 Hz, 1H), 7.78–7.72 (m, 2H), 7.58 (d, *J* = 8.1 Hz, 1H), 7.50 (dd, *J* = 8.1, 2.1 Hz, 1H), 4.11 (s, 2H); ^13^C NMR (150 MHz, CDCl_3_) δ: 159.28, 153.47, 142.06, 141.52, 141.47, 139.71, 134.32, 131.04, 129.47, 129.34, 129.25, 129.00, 126.92, 122.71, 35.60; HRMS (ESI) calcd for C_15_H_10_ClN_2_^+^ (M + H)^+^ 253.0527, found 253.0519.

2-chloro-7,8-dimethyl-11*H*-indeno[1,2-b]quinoxaline (**2.3bb-1**)

White solid; 36% yield; ^1^H NMR (600 MHz, CDCl_3_) δ: 8.16 (d, *J* = 2.0 Hz, 1H), 7.88 (s, 1H), 7.80 (s, 1H), 7.54 (d, *J* = 8.0 Hz, 1H), 7.45 (dd, *J* = 8.1, 2.0 Hz, 1H), 4.04 (s, 2H), 2.49 (d, *J* = 3.0 Hz, 6H); ^13^C NMR (150 MHz, CDCl_3_) δ: 158.27, 152.50, 141.25, 140.82, 140.33, 140.06, 139.79, 139.71, 134.12, 130.47, 128.48, 128.17, 126.78, 122.35, 35.54, 20.32, 20.27; HRMS (ESI) calcd for C_17_H_14_ClN_2_^+^ (M + H)^+^ 281.0840, found 281.0833.

3-chloro-7,8-dimethyl-11*H*-indeno[1,2-b]quinoxaline (**2.3bb-2**)

White solid; 40% yield; ^1^H NMR (600 MHz, Chloroform-*d*) δ: 8.10 (d, *J* = 8.1 Hz, 1H), 7.87 (s, 1H), 7.79 (s, 1H), 7.59 (d, *J* = 1.8 Hz, 1H), 7.46 (dd, *J* = 8.1, 1.9 Hz, 1H), 4.05 (s, 2H), 2.49 (s, 6H); ^13^C NMR (150 MHz, CDCl_3_) δ: 157.83, 152.59, 144.64, 140.76, 140.09, 139.73, 139.48, 136.88, 136.53, 128.43, 128.37, 128.19, 125.99, 123.30, 35.73, 20.30, 20.26; HRMS (ESI) calcd for C_17_H_14_ClN_2_^+^ (M + H)^+^ 281.0840, found 281.0834.

### 3.3. Recycle of β-CD after Synthesis

After completion of the reaction, the mixture was extracted by ethyl acetate (2 × 10 mL) and dried over anhydrous sodium sulfate. Ethyl acetate was removed in a vacuum and the crude product was obtained. Then, the water layer was recycled and removed in a vacuum to obtain the reused catalyst.

## 4. Conclusions

In conclusion, we developed a simple one-pot method for the synthesis of indeno[1,2-b]quinoxaline derivatives from the reaction of 2-indanones with OPDs using *β*-CD as a catalyst in water. This method has the advantages of high-yield, low-cost, biomimetic, and neutral aqueous-phase conditions. It is environmentally friendly; moreover, the catalyst is easy to recover and to reuse. Therefore, this straightforward method may find widespread applications in medicinal and organic chemistry.

## Figures and Tables

**Table 1:** Optimization of reaction conditions in the synthesis of indeno[1,2-b]quinoxaline from 2-indanone and OPD *^a^*.

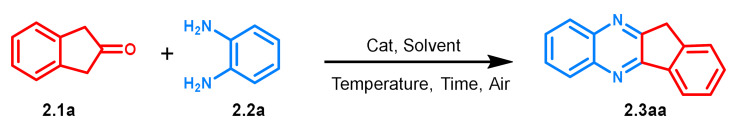						
Entry	2.2a/2.1a	Cat	Solvent	Temperature (°C)	Time (h)	Yield *^c^* (2.3)
1	1.5	No Catalyst	H_2_O	R.T	24	Trace
2	1.5	*α*-CD (20 Mol%)	H_2_O	R.T	24	61
3	1.5	*β*-CD (20 Mol%)	H_2_O	R.T	24	85
4	1.5	*γ*-CD (20 Mol%)	H_2_O	R.T	24	52
5	1.5	Trimethyl-*β*-CD (20 Mol%)	H_2_O	R.T	24	38
6	1.5	2,6-Dimethyl-*β*-CD (20 Mol%)	H_2_O	R.T	24	56
7	1.5	Me-*β*-CD	H_2_O	R.T	24	53
8	1.5	NEt_3_ (20 Mol%)	H_2_O	60	24	33
9	1.5	*β*-CD (20 Mol%, grind)	None	R.T	0.5	70
10 *^b^*	1.5	*β*-CD (20 Mol%)	H_2_O	R.T	24	Trace
11	1.5	*β*-CD (20 Mol%)	DMSO	R.T	24	73
12	1.5	*β*-CD (20 Mol%)	DMF	R.T	24	76
13	1.5	*β*-CD (20 Mol%)	EtOH	R.T	24	67
14	1.5	*β*-CD (20 Mol%)	H_2_O	60	24	43
15	1.5	*β*-CD (15 Mol%)	H_2_O	R.T	12	85
16	1.2	*β*-CD (15 Mol%)	H_2_O	R.T	12	85
17	1.0	*β*-CD (15 Mol%)	H_2_O	R.T	12	81

*^a^* Standard reaction conditions: **2.1a** (0.2 mmol), **2.2a** (1.0–1.5 eq) with various reaction conditions in air. *^b^* Instead of air in a nitrogen atmosphere. *^c^* Isolated yields. R.T = room temperature (25 °C).

## Data Availability

The data presented in this study are available in insert article or Supplementary Materials here.

## Supplementary Materials

The following are available online, Figure S1: ^1^H NMR spectrum of compound **2.3aa**; Figure S2: ^1^^3^C NMR spectrum of compound **2.3aa**; Figure S3: ^1^H NMR spectrum of compounds **2.3ab-1** and **2.3ab-2**; Figure S4: ^1^^3^C NMR spectrum of compounds **2.3ab-1** and **2.3ab-2**; Figure S5: ^1^H NMR spectrum of compound **2.3ac-1**; Figure S6: ^1^^3^C NMR spectrum of compound **2.3ac-1**; Figure S7: ^1^H NMR spectrum of compound **2.3ac-2**; Figure S8: ^1^^3^C NMR spectrum of compound **2.3ac-2**; Figure S9: ^1^H NMR spectrum of compound **2.3ad-1**; Figure S10: ^1^^3^C NMR spectrum of compound **2.3ad-1**; Figure S11: ^1^H NMR spectrum of compound **2.3ad-2**; Figure S12: ^1^^3^C NMR spectrum of compound **2.3ad-2**; Figure S13: ^1^H NMR spectrum of compound **2.3ae-1**; Figure S14: ^1^^3^C NMR spectrum of compound **2.3ae-1**; Figure S15: ^1^H NMR spectrum of compound **2.3ae-2**; Figure S16: ^1^^3^C NMR spectrum of compound **2.3ae-2**; Figure S17: ^1^H NMR spectrum of compound **2.3af**; Figure S18: ^1^^3^C NMR spectrum of compound **2.3af**; Figure S19: ^1^H NMR spectrum of compound **2.3ag**; Figure S20: ^1^^3^C NMR spectrum of compound **2.3ag**; Figure S21: ^1^H NMR spectrum of compound **2.3ah**; Figure S22: ^1^^3^C NMR spectrum of compound **2.3ah**; Figure S23: ^1^H NMR spectrum of compound **2.3ai**; Figure S24: ^1^^3^C NMR spectrum of compound **2.3ai**; Figure S25: ^1^H NMR spectrum of compound **2.3aj**; Figure S26: ^1^^3^C NMR spectrum of compound **2.3aj**; Figure S27: ^1^H NMR spectrum of compound **2.3ba-1**; Figure S28: ^1^^3^C NMR spectrum of compound **2.3ba-1**; Figure S29: ^1^H NMR spectrum of compound **2.3ba-2**; Figure S30: ^1^^3^C NMR spectrum of compound **2.3ba-2**; Figure S31: ^1^H NMR spectrum of compound **2.3bb-1**; Figure S32: ^1^^3^C NMR spectrum of compound **2.3bb-1**; Figure S33: ^1^H NMR spectrum of compound **2.3bb-2**; Figure S34: ^1^^3^C NMR spectrum of compound **2.3bb-2**; Figure S35: Mass spectrum smartFormula report of compound **2.3aa**; Figure S36: Mass spectrum smartFormula report of compounds **2.3ab-1** and **2.3ab-2**; Figure S37: Mass spectrum smartFormula report of compound **2.3ac-1**; Figure S38: Mass spectrum smartFormula report of compound **2.3ac-2**; Figure S39: Mass spectrum smartFormula report of compound **2.3ad-1**; Figure S40: Mass spectrum smartFormula report of compound **2.3ad-2**; Figure S41: Mass spectrum smartFormula report of compound **2.3ae-1**; Figure S42: Mass spectrum smartFormula report of compound **2.3ae-2**; Figure S43: Mass spectrum smartFormula report of compound **2.3af**; Figure S44: Mass spectrum smartFormula report of compound **2.3ag**; Figure S45: Mass spectrum smartFormula report of compound **2.3ah**; Figure S46: Mass spectrum smartFormula report of compound **2.3ai**; Figure S47: Mass spectrum smartFormula report of compound **2.3aj**; Figure S48: Mass spectrum smartFormula report of compound **2.3ba-1**; Figure S49: Mass spectrum smartFormula report of compound **2.3ba-2**; Figure S50: Mass spectrum smartFormula report of compound **2.3bb-1**; Figure S51: Mass spectrum smartFormula report of compound **2.3bb-2**; Figure S52: The NOESY spectrum of 2-indenone (1.0 eq.) and *β*-CD (15 mol%) mixed after stirring in water for 12 h; Figure S53: The NOESY spectrum of OPD (1.2 eq.) and *β*-CD (15 mol%) mixed after stirring in water for 12 h; Figure S54: The NOESY spectrum of 2,3-diaminonaphthalene (1.2 eq.) and *β*-CD (15 mol%) mixed after stirring in water for 12 h; Figure S55: The HRMS of template reaction (2.1a (0.2 mmol), 2.2a (0.24 mmol) and β-CD (15 %) in air) in water after 1 h.

## Sample Availability

Samples of the compounds are not available from the authors.
