# Supplementary material for: Green Synthesis of Indeno[1,2-b]quinoxalines Using β-Cyclodextrin as Catalyst
Source: Molecules. 2022 Jan 17;27(2):580. doi: 10.3390/molecules27020580 (PMC8779894; doi:10.3390/molecules27020580)
Supplement: Supplementary file 1 [file molecules-27-00580-s001.zip › molecules-1536384-supplementary.pdf]

Supplementary Materials

The green synthesis of indeno[1,2-b]quinoxalines using *β*-Cyclodextrin as catalyst

Li-Guo Liao [1,2], Meng-Meng Song [3], Jun-Feng Feng [3], Min Tan [1,2], Fan Liu [1,2], Zhen-jiang Qiu [1,2], Sheng Zhang [3,] and Bang-jing Li [1,2,]**

[1] Key Laboratory of Mountain Ecological Restoration and Bioresource Utilization, Chengdu Institute of Biology, Chinese Academy of Sciences, Chengdu 610041, China

[2] University of Chinese Academy of Sciences, Beijing 100049, China

[3] State Key Laboratory of Polymer Materials Engineering, Polymer Research Institute of Sichuan University, Chengdu 610065, China

** Corresponding authors: zhangsheng@scu.edu.cn (S. Zhang), libj@cib.ac.cn (B.-J. Li)*

CONTENTS

1.. NMR spectra

Copies of ^{1}H NMR and ^{13}C NMR spectra of **2.3aa-bb**……………….....……….......**2-18**

2.. HRMS spectra

Copies of Mass Spectrum SmartFormula Report of **2.3aa-bb**……….....………........**19-27**

3..NOESY spectra

Copies of the NOESY spectrums of 2**.1a** mixed with *β*-CD……….....……..............**27**

Copies of the NOESY spectrums of 2**.2a** mixed with *β*-CD……….....……..............**28**

Copies of the NOESY spectrums of 2**.2j** mixed with *β*-CD……….....……..............**28**

4.. HRMS spectra

Copies of the HRMS of template reaction after 1h......………...……........................**29**

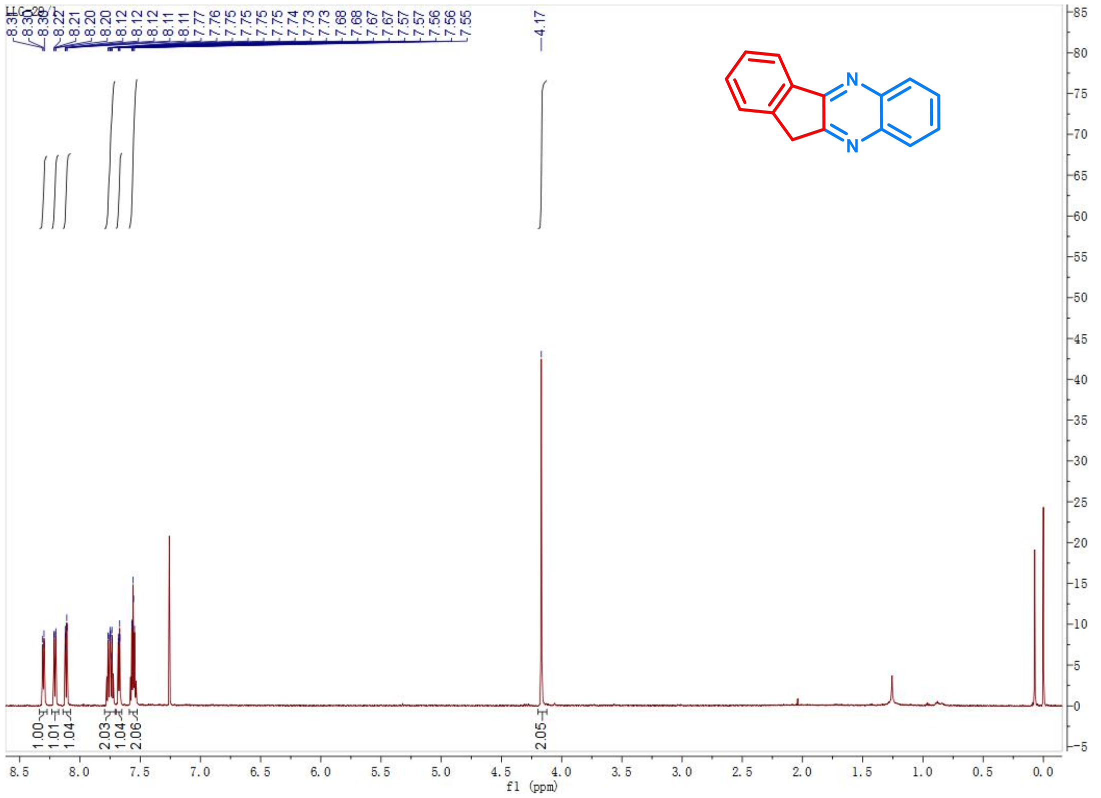

Figure S1. [1] H NMR spectrum of compound **2.3aa**.

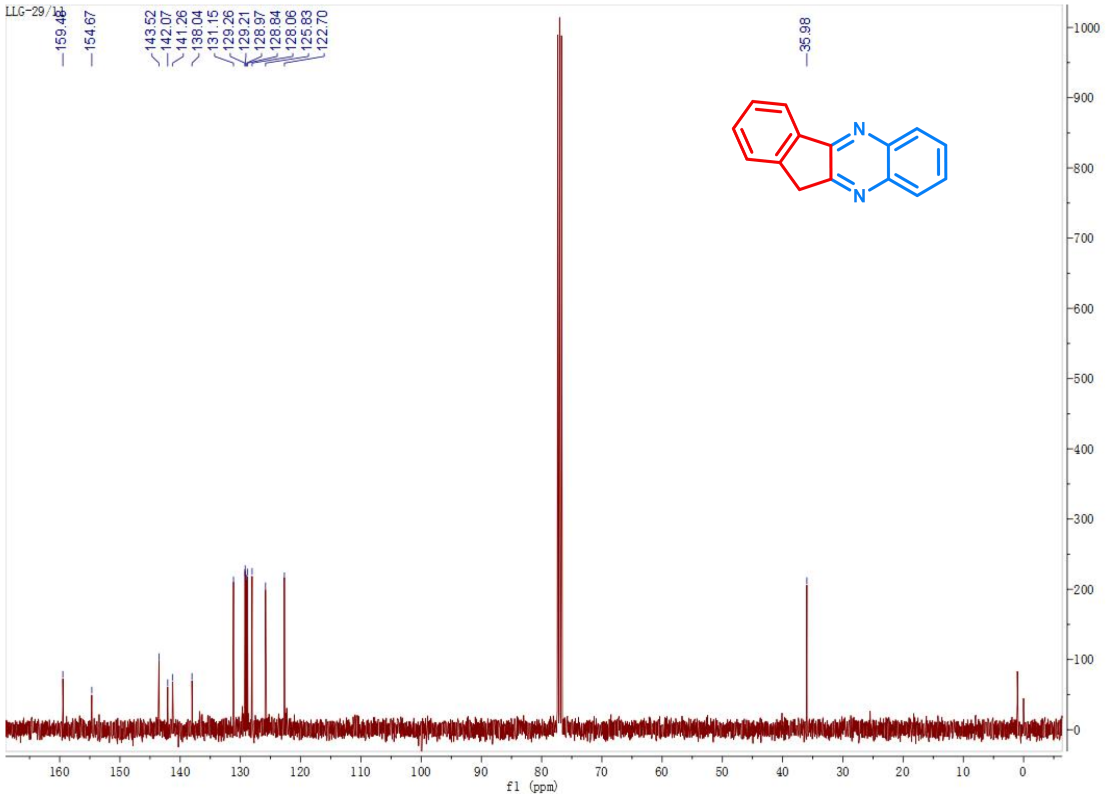

Figure S2. [13] C NMR spectrum of compound **2.3aa**.

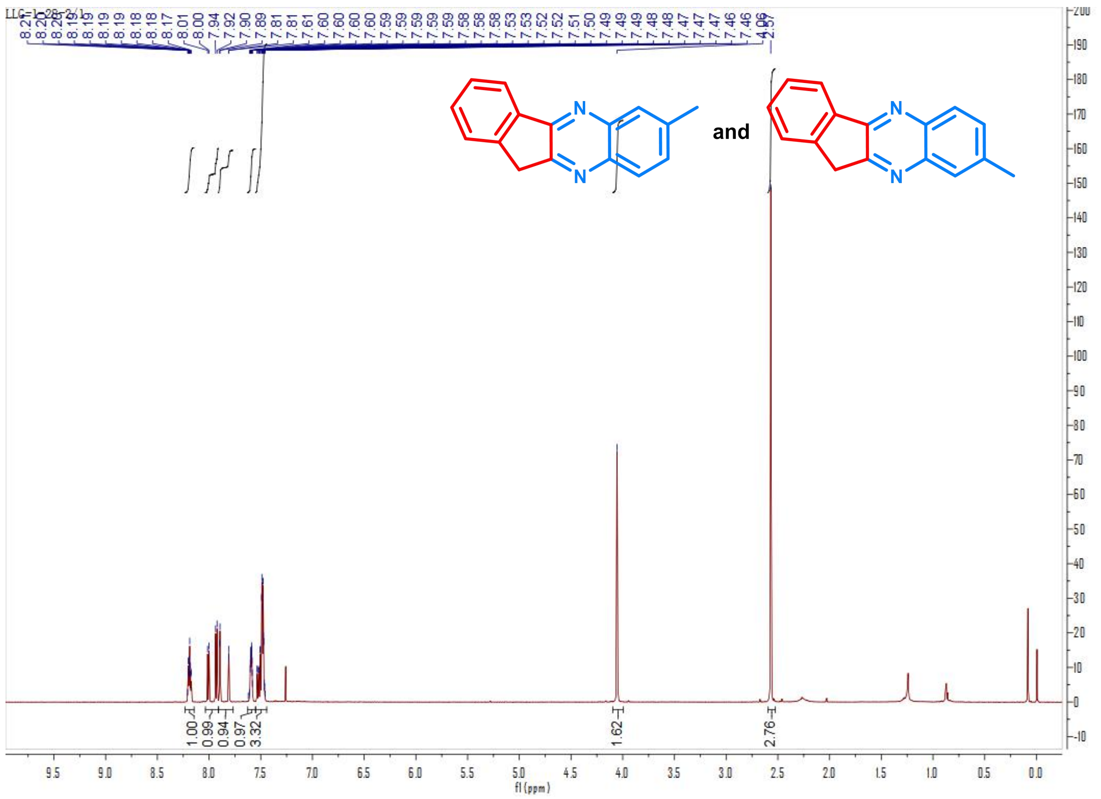

Figure S3. [1] H NMR spectrum of compounds **2.3ab-1** and **2.3ab-2**.

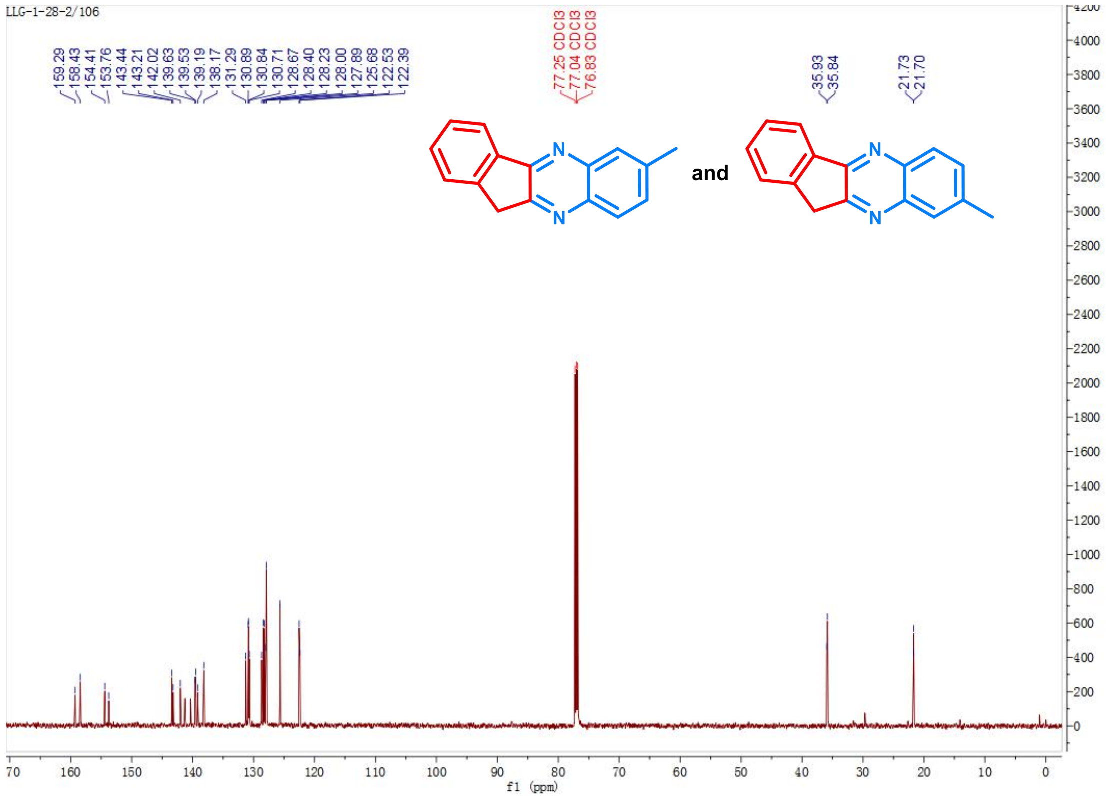

Figure S4. [13] C NMR spectrum of compounds **2.3ab-1** and **2.3ab-2**.

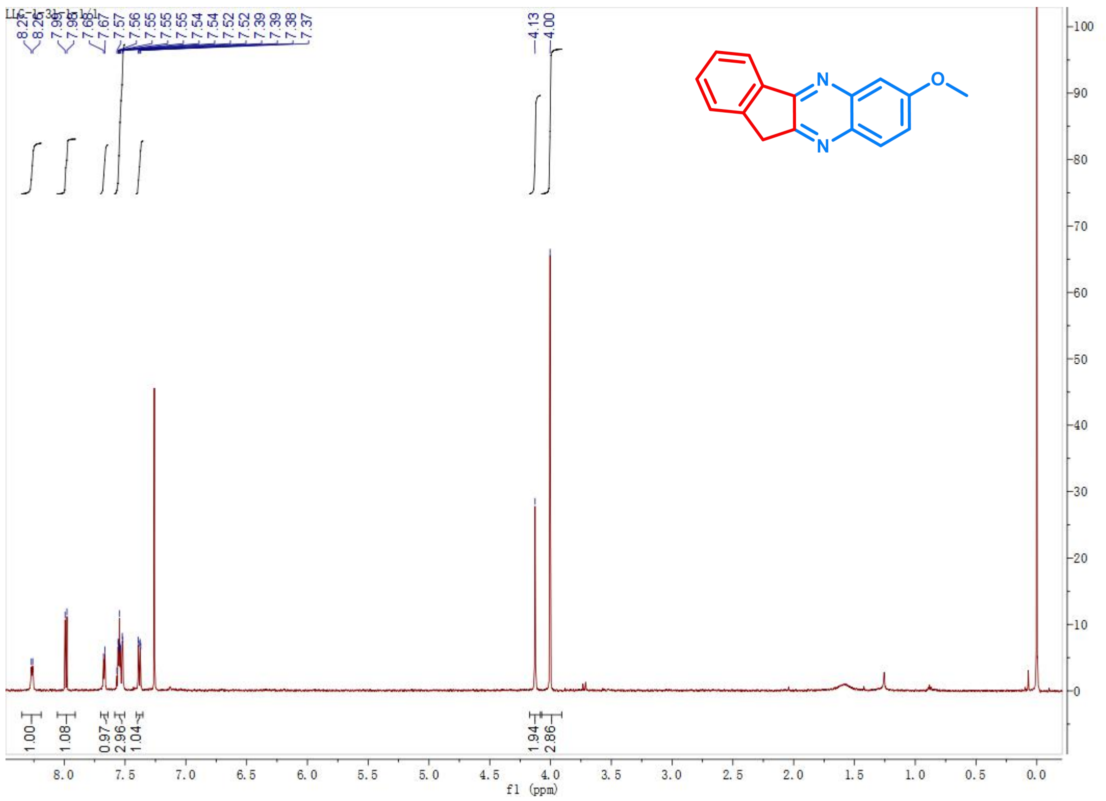

Figure S5. ^{1}H NMR spectrum of compound **2.3ac-1**.

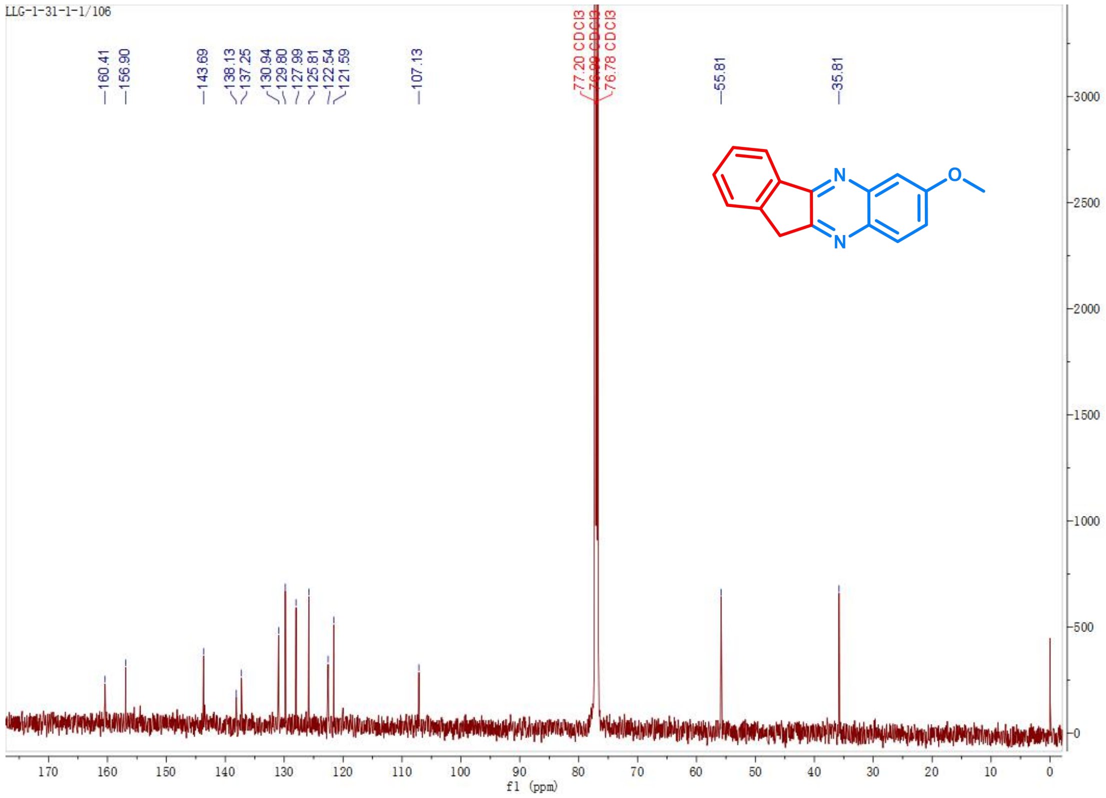

Figure S6. ^{13}C NMR spectrum of compound **2.3ac-1**.

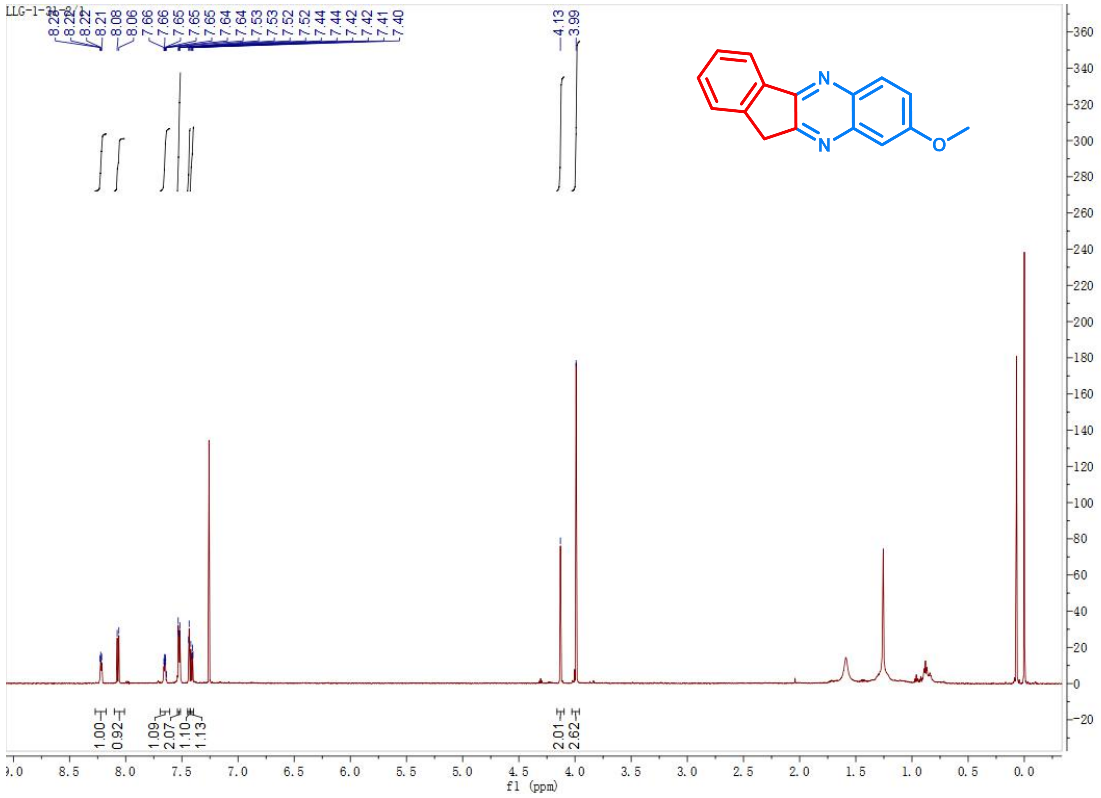

Figure S7. [1] H NMR spectrum of compound **2.3ac-2**.

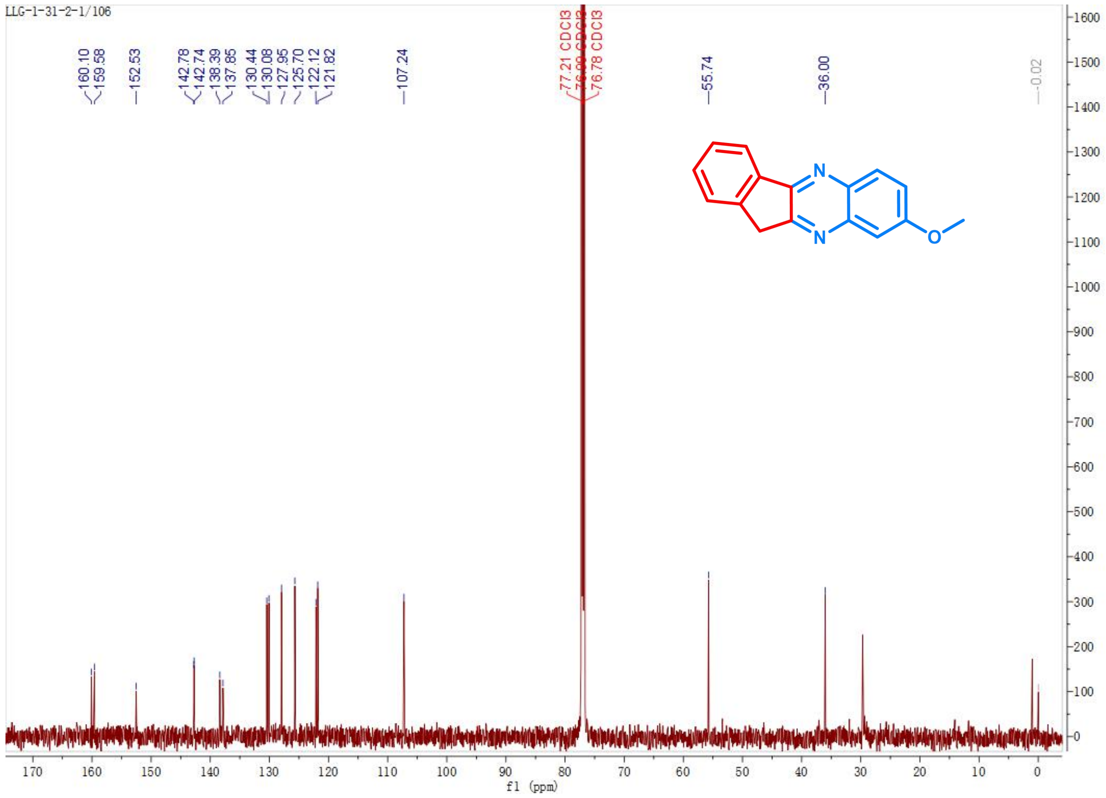

Figure S8. [13] C NMR spectrum of compound **2.3ac-2**.

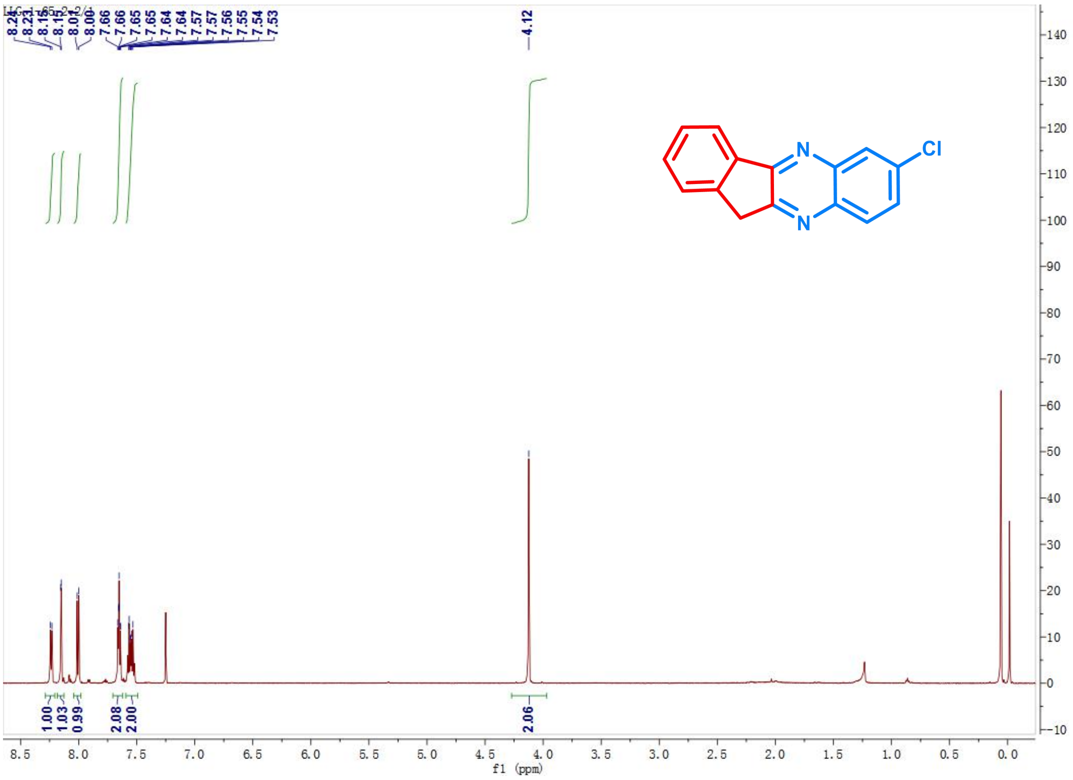

Figure S9. [1] H NMR spectrum of compound **2.3ad-1**.

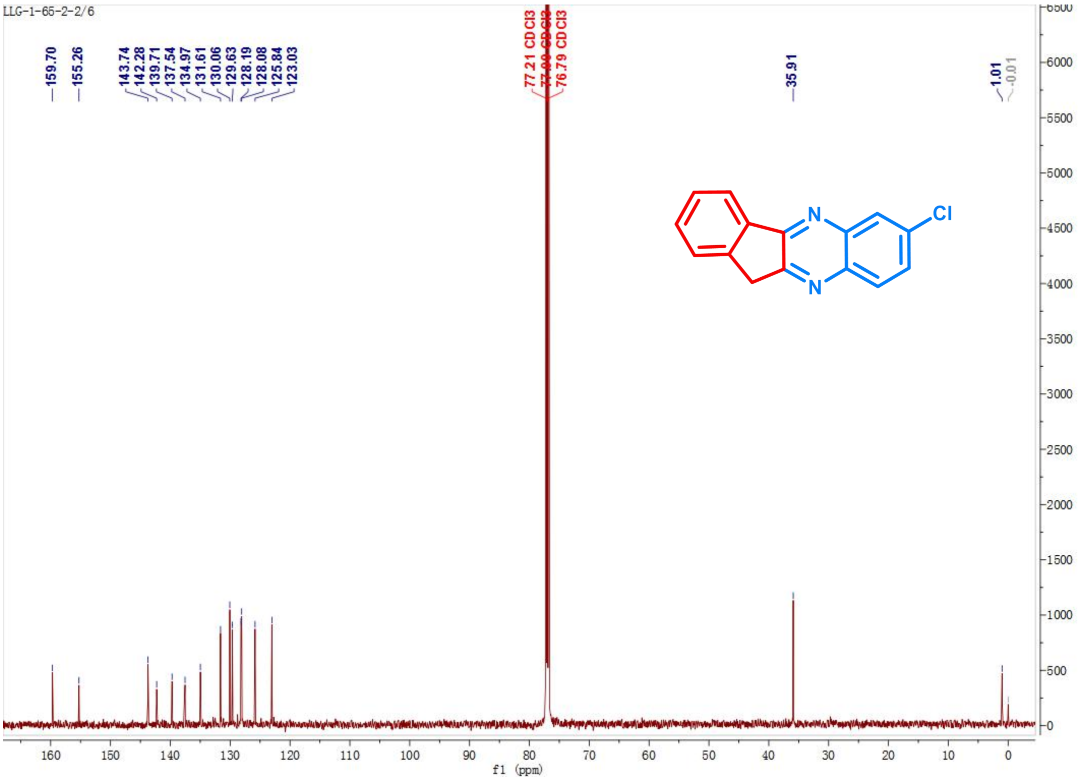

Figure S10. [13] C NMR spectrum of compound **2.3ad-1**.

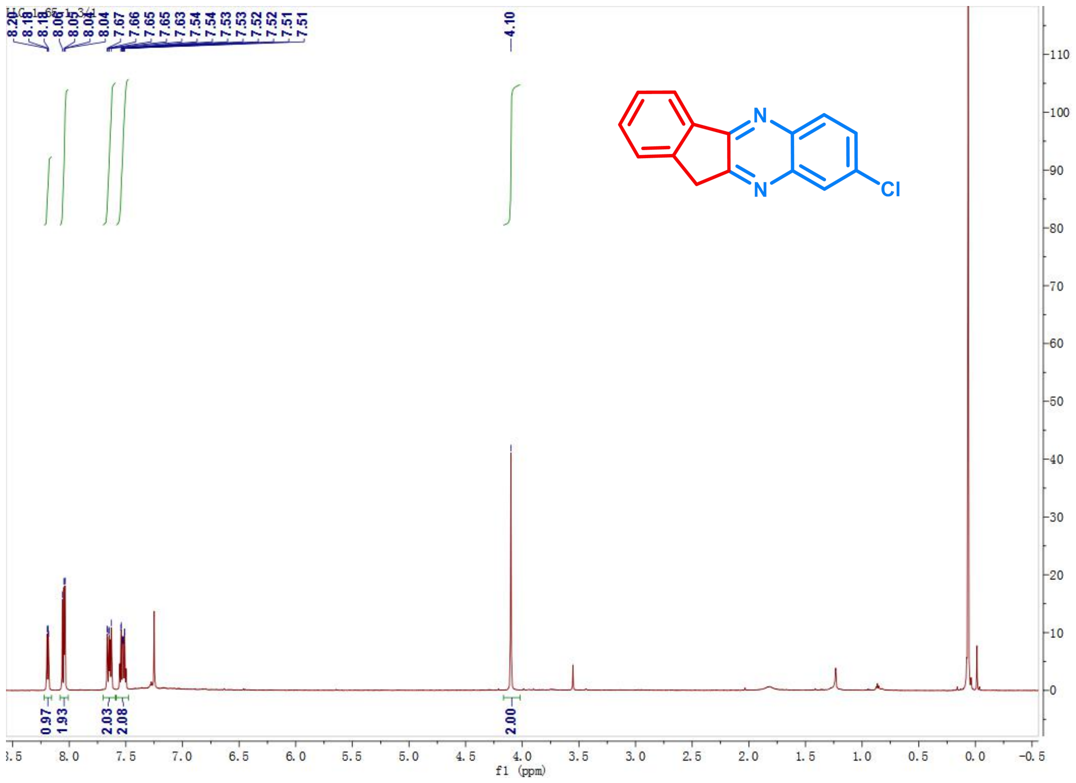

Figure S11. [1] H NMR spectrum of compound **2.3ad-2**.

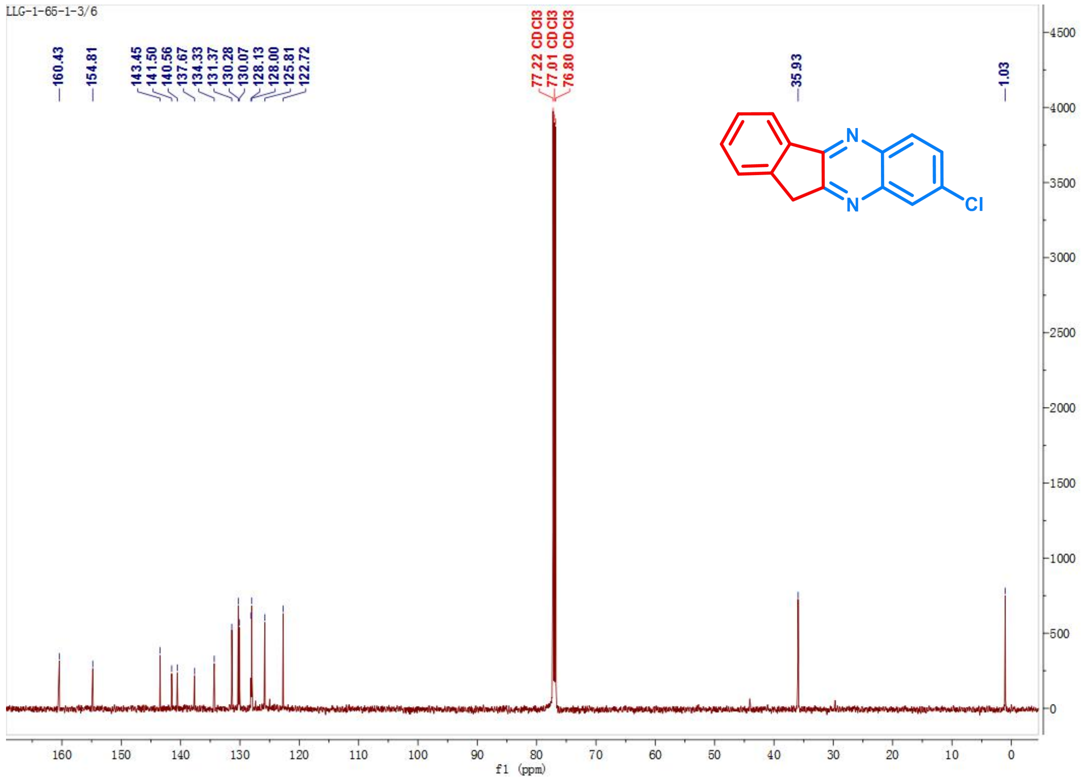

Figure S12. [13] C NMR spectrum of compound **2.3ad-2**.

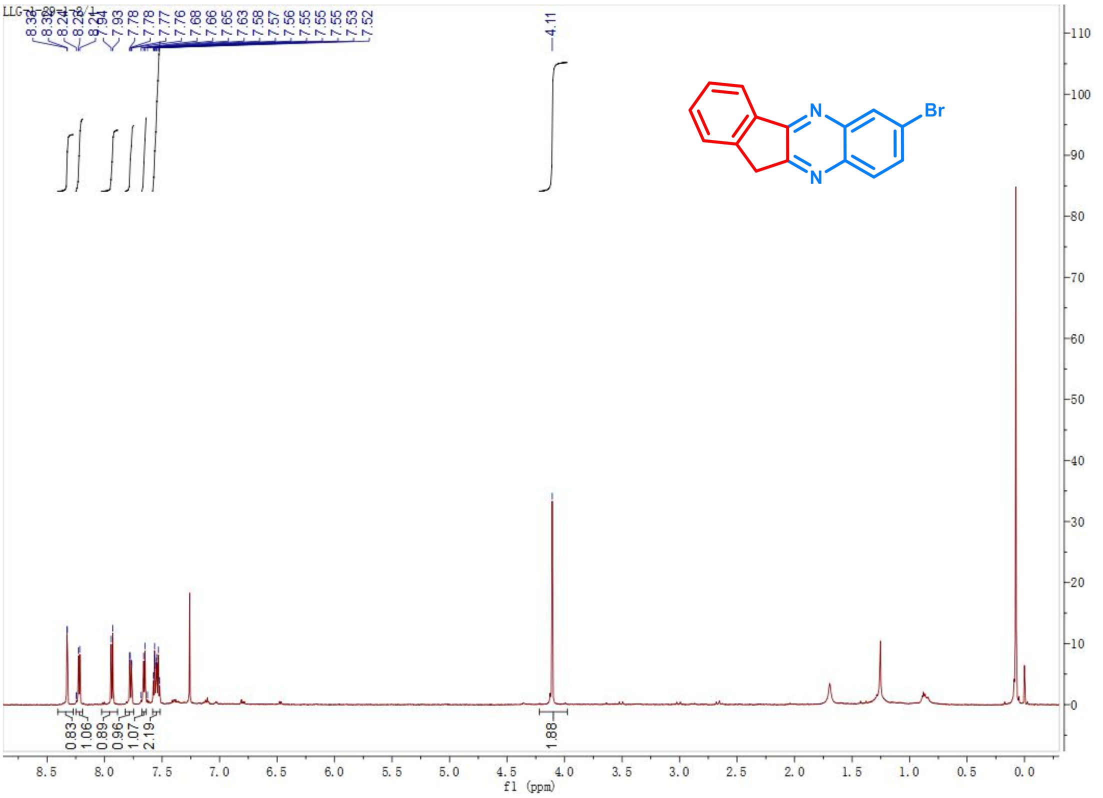

Figure S13. [1] H NMR spectrum of compound **2.3ae-1**.

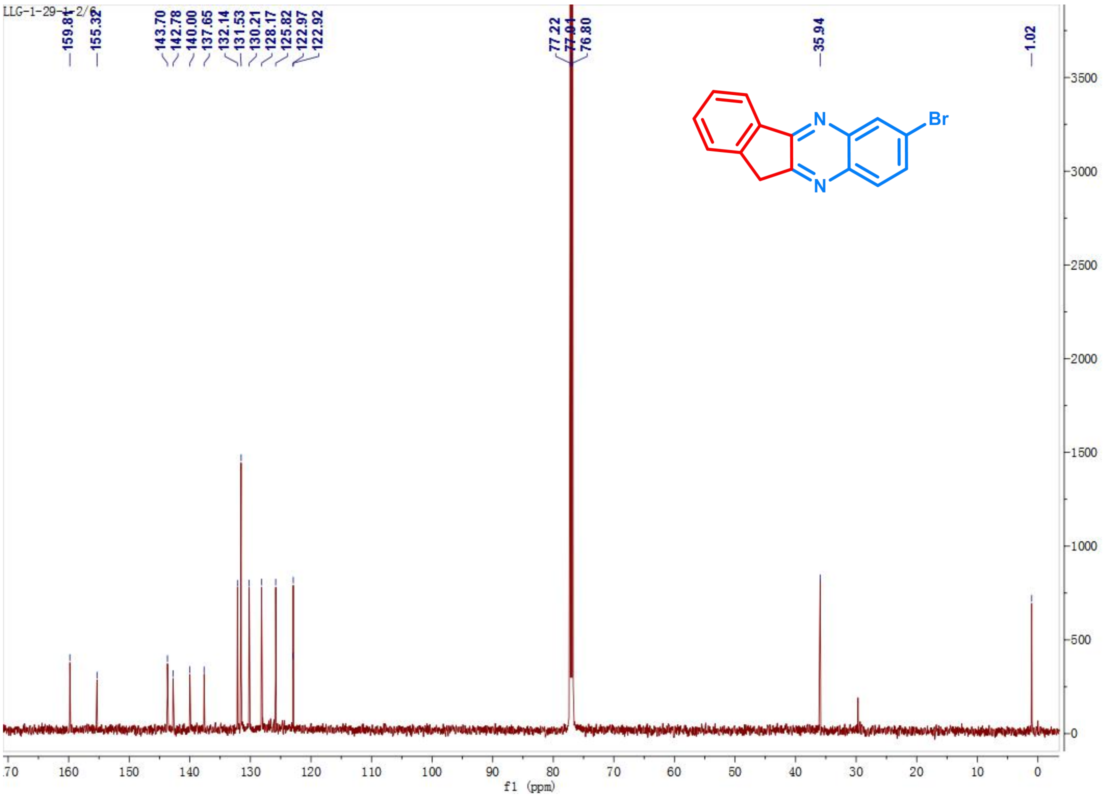

Figure S14. [13] C NMR spectrum of compound **2.3ae-1**.

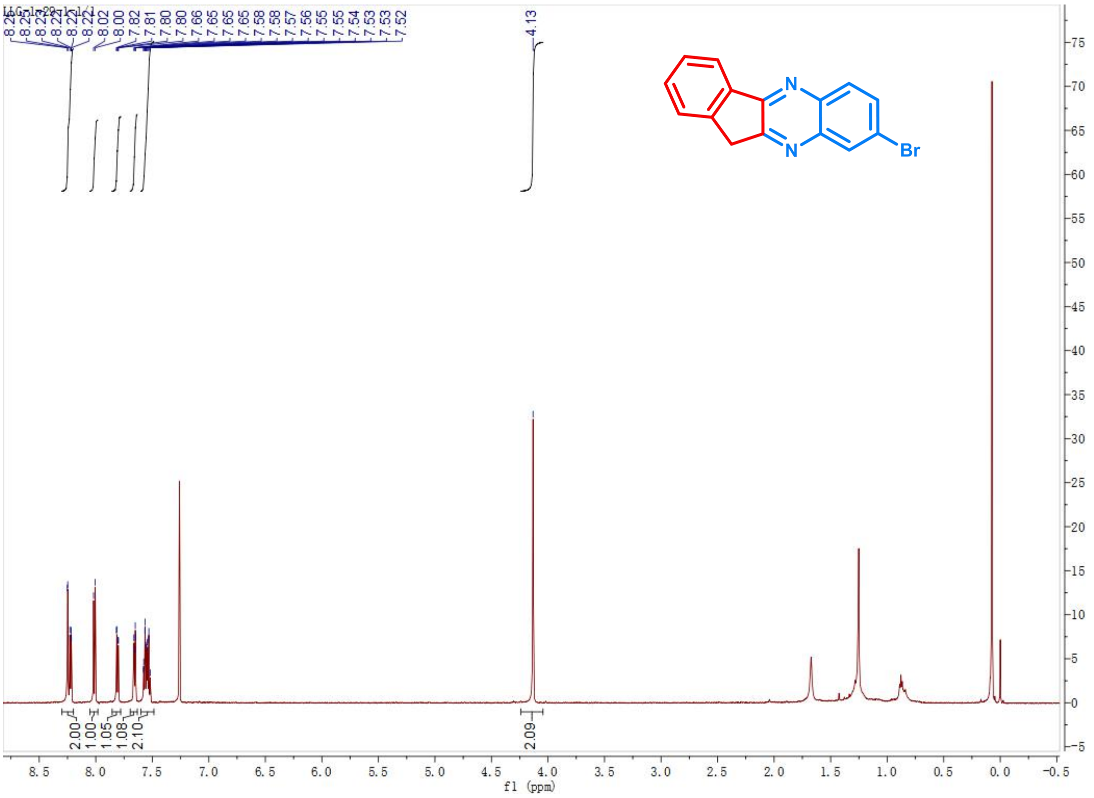

Figure S15. ^{1}H NMR spectrum of compound **2.3ae-2**.

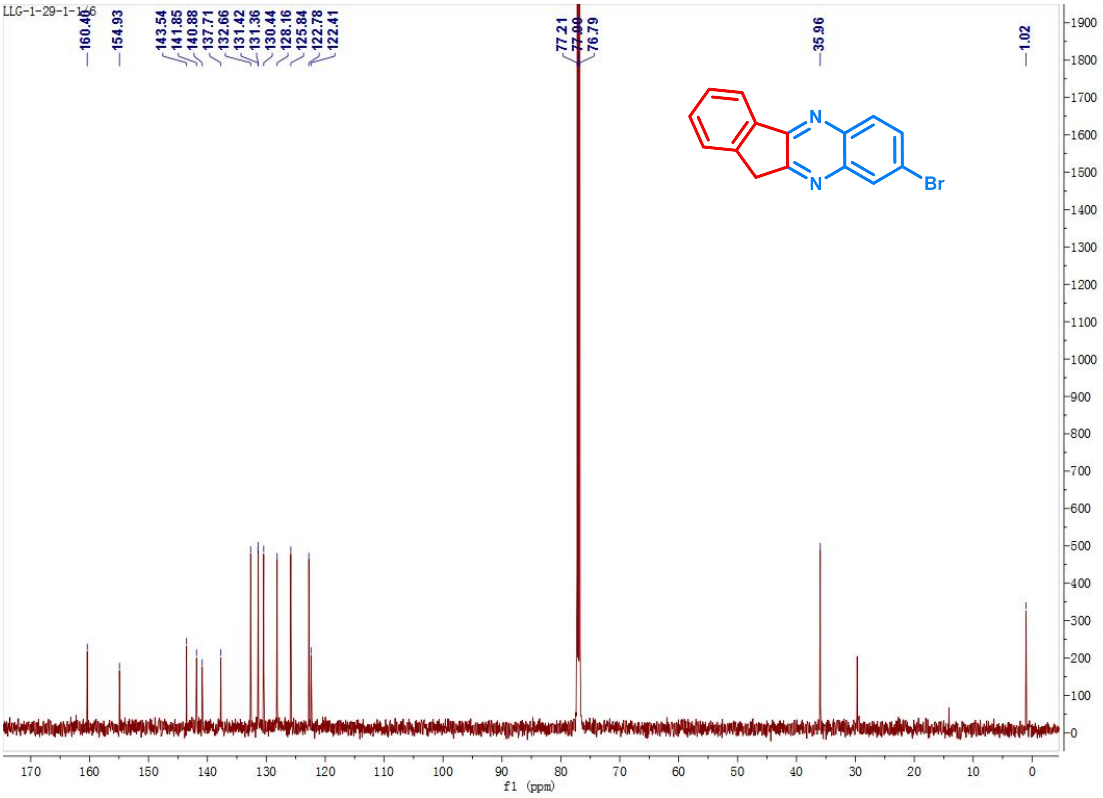

Figure S16. ^{13}C NMR spectrum of compound **2.3ae-2**.

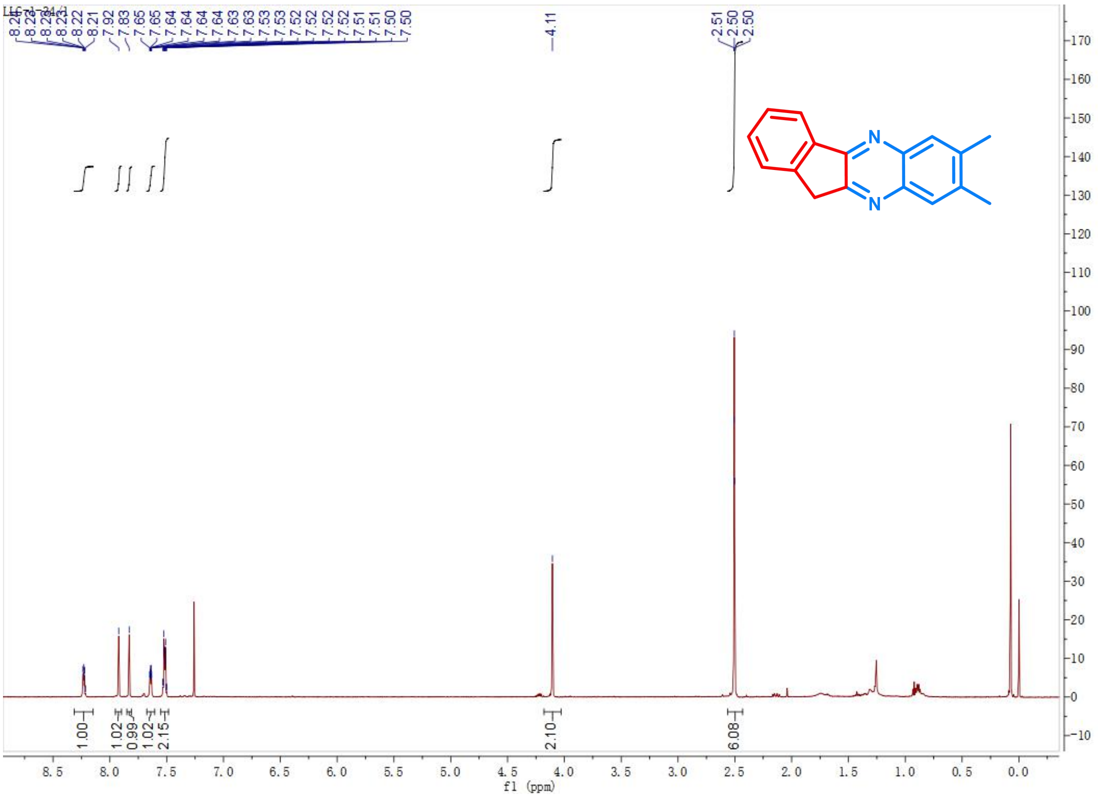

Figure S17. [1] H NMR spectrum of compound **2.3af**.

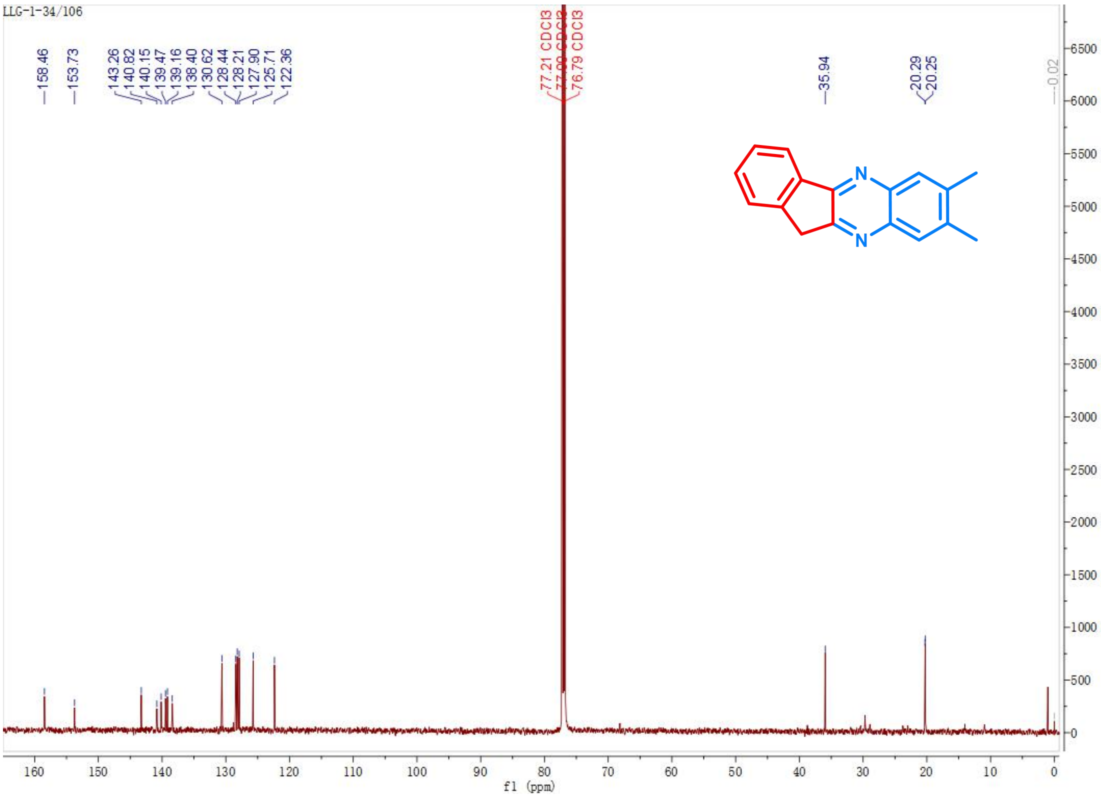

Figure S18. [13] C NMR spectrum of compound **2.3af**.

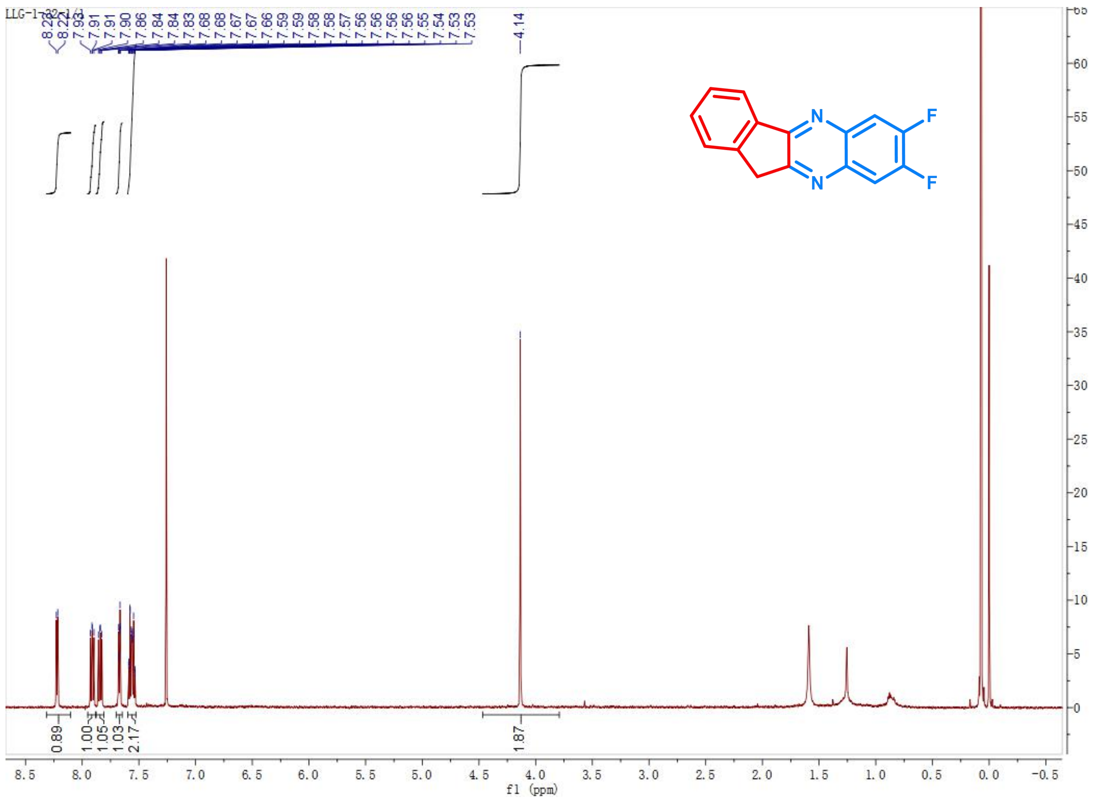

Figure S19. [1] H NMR spectrum of compound **2.3ag**.

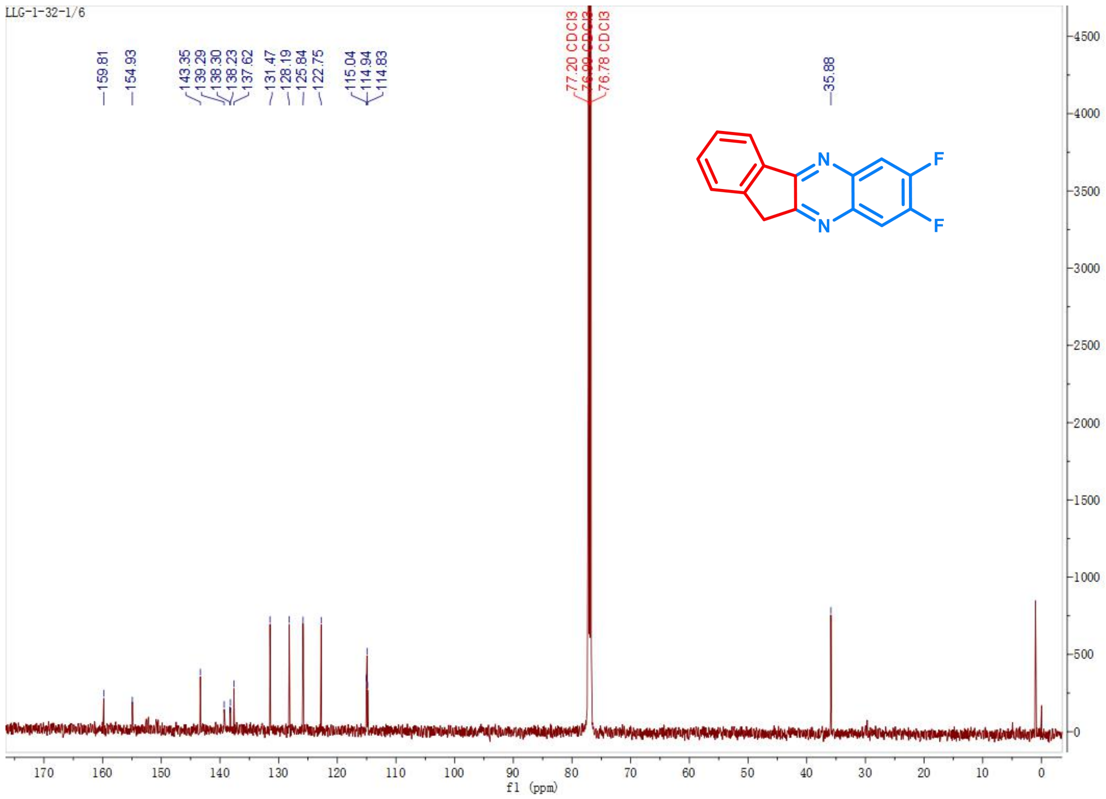

Figure S20. [13] C NMR spectrum of compound **2.3ag**.

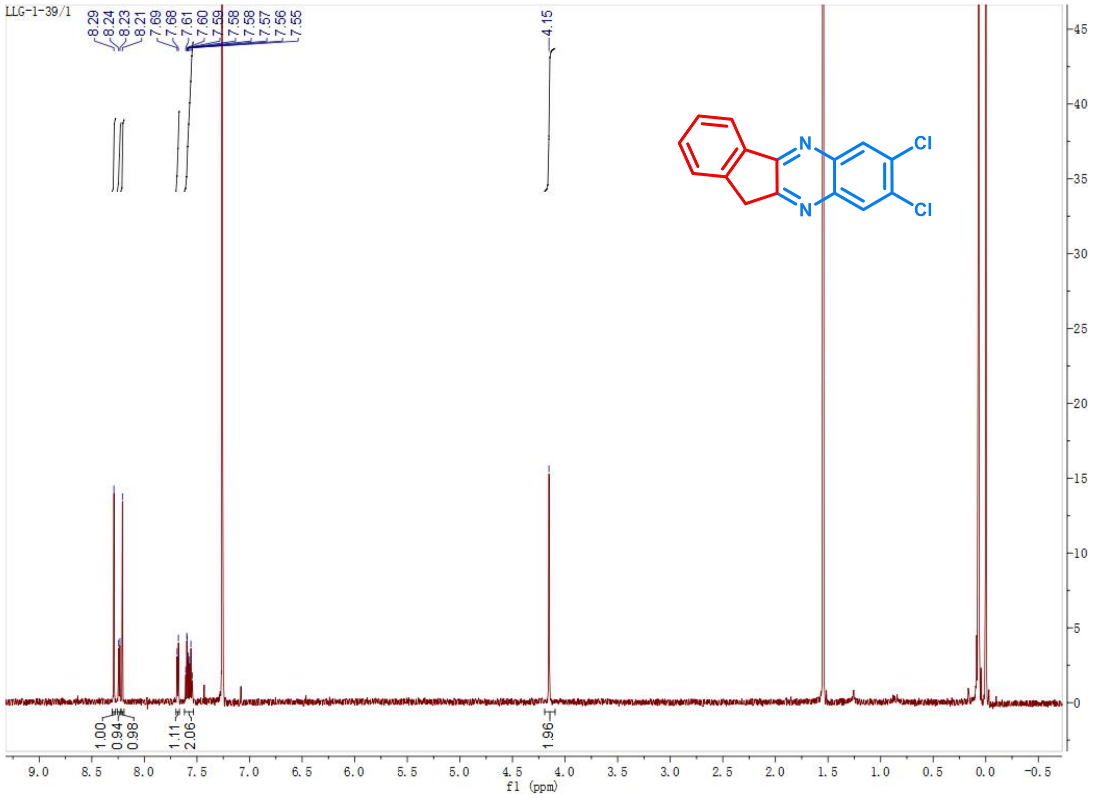

Figure S21. [1] H NMR spectrum of compound **2.3ah**.

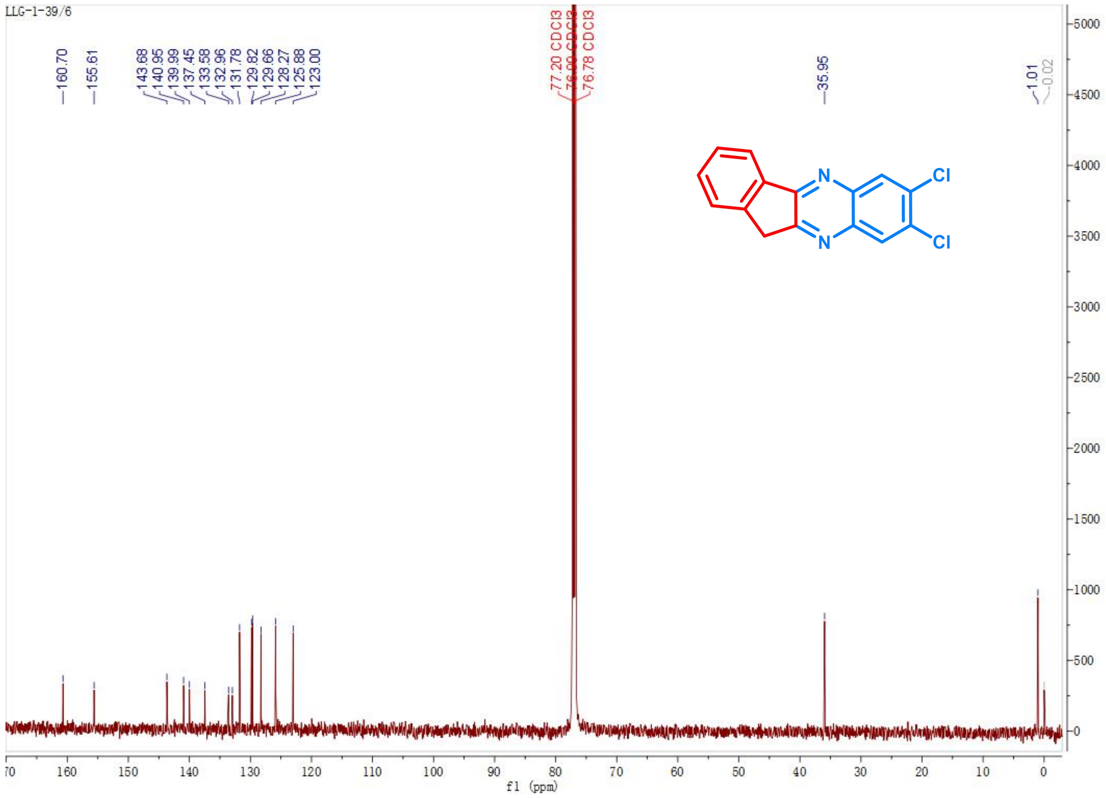

Figure S22. [13] C NMR spectrum of compound **2.3ah**.

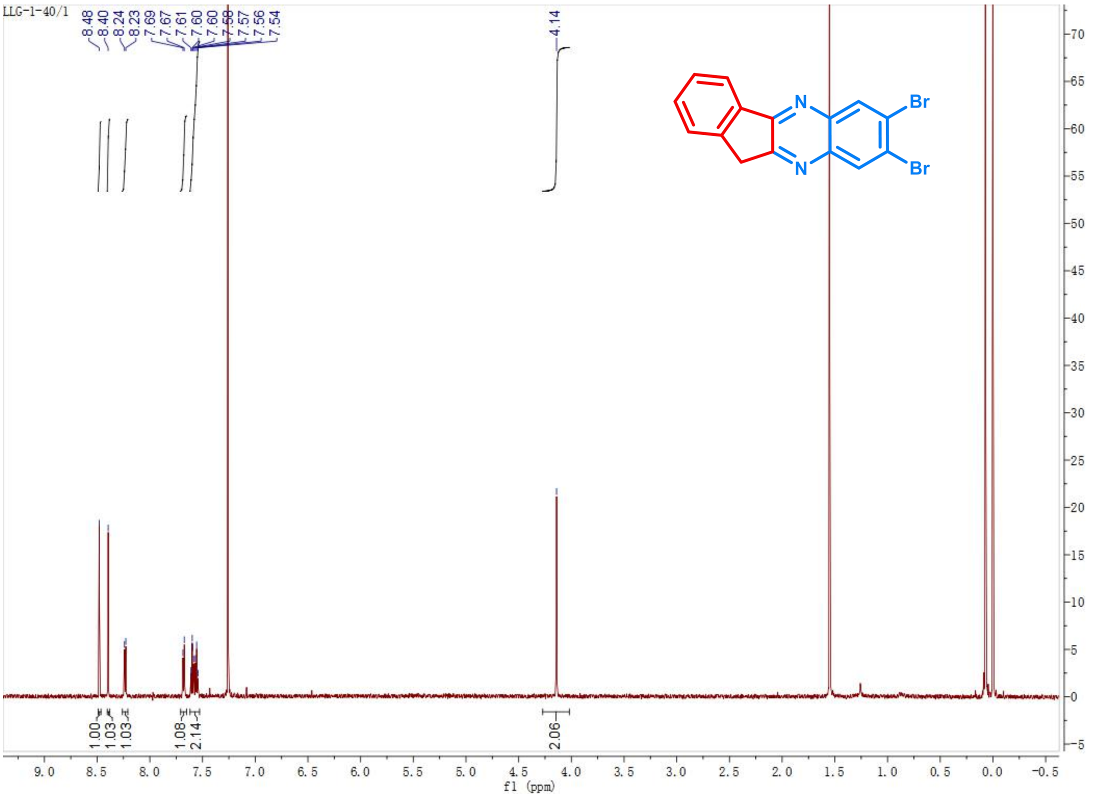

Figure S23. [1] H NMR spectrum of compound **2.3ai**.

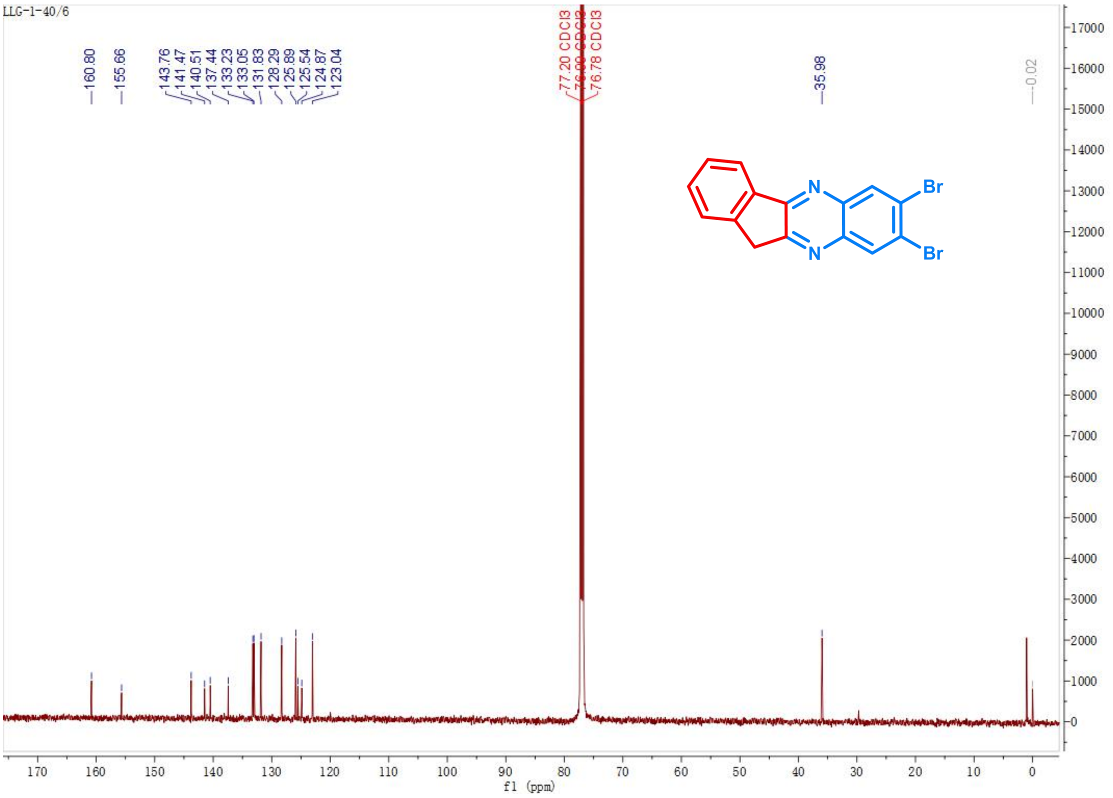

Figure S24. [13] C NMR spectrum of compound **2.3ai**.

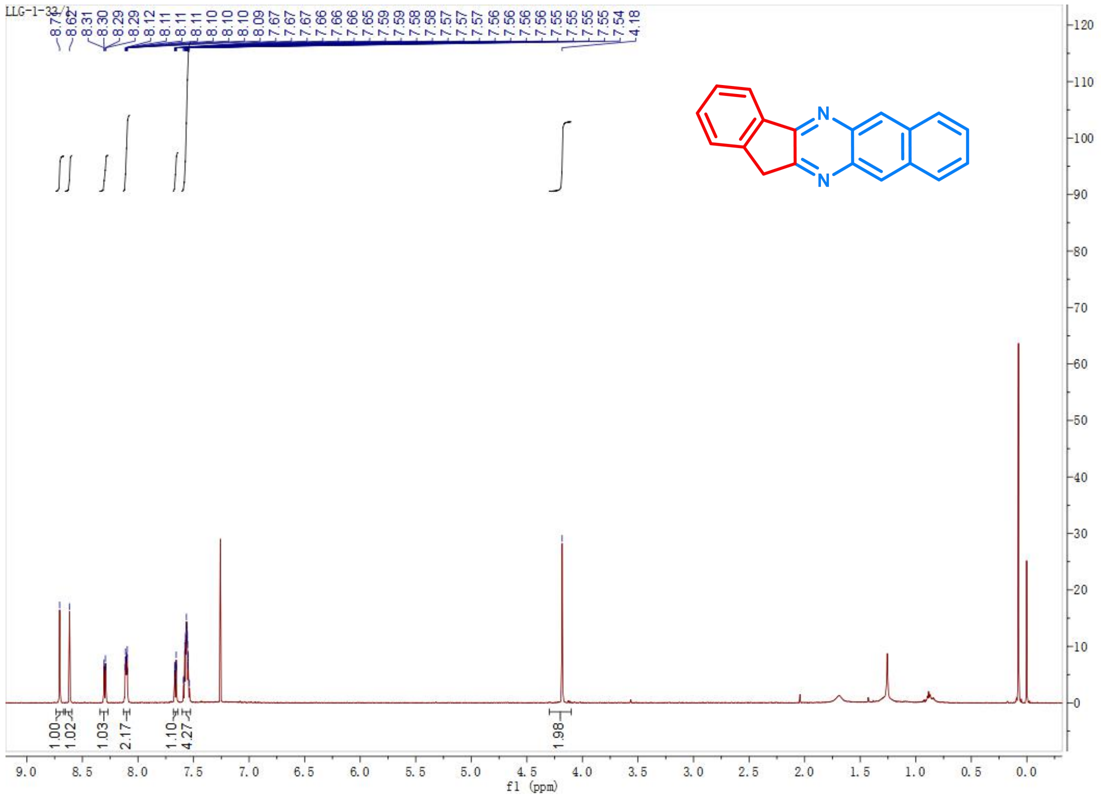

Figure S25. [1] H NMR spectrum of compound **2.3aj**.

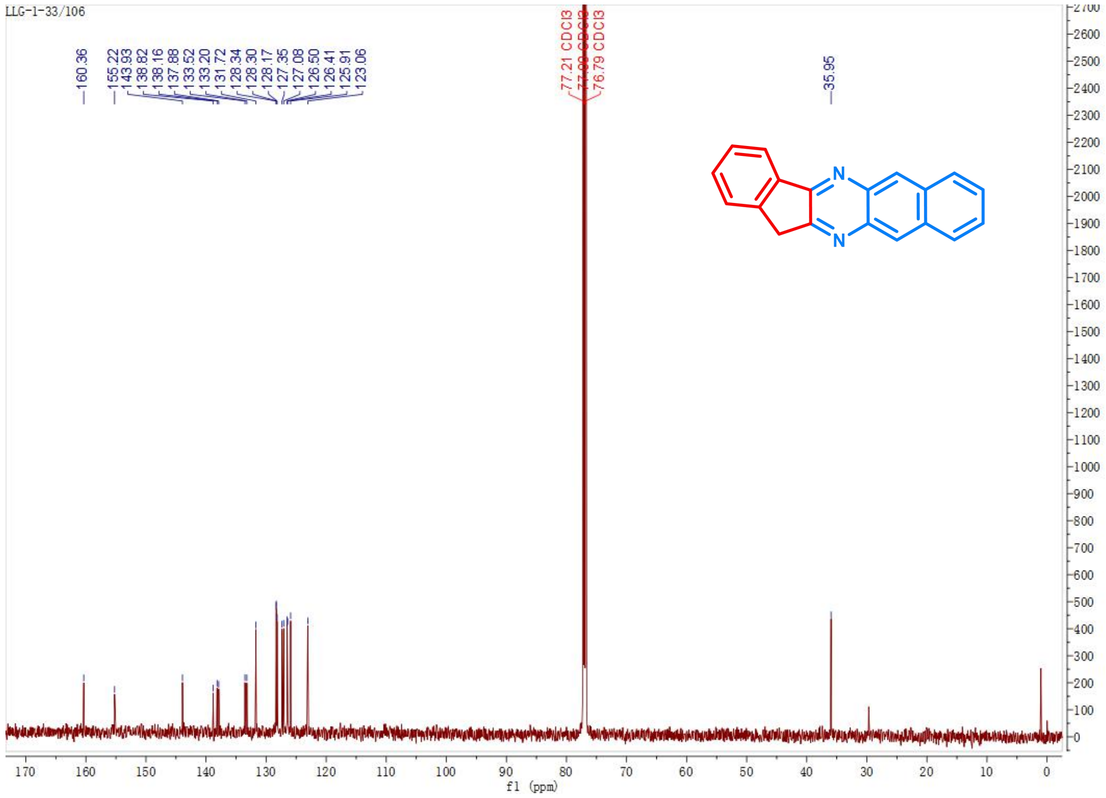

Figure S26. [13] C NMR spectrum of compound **2.3aj**.

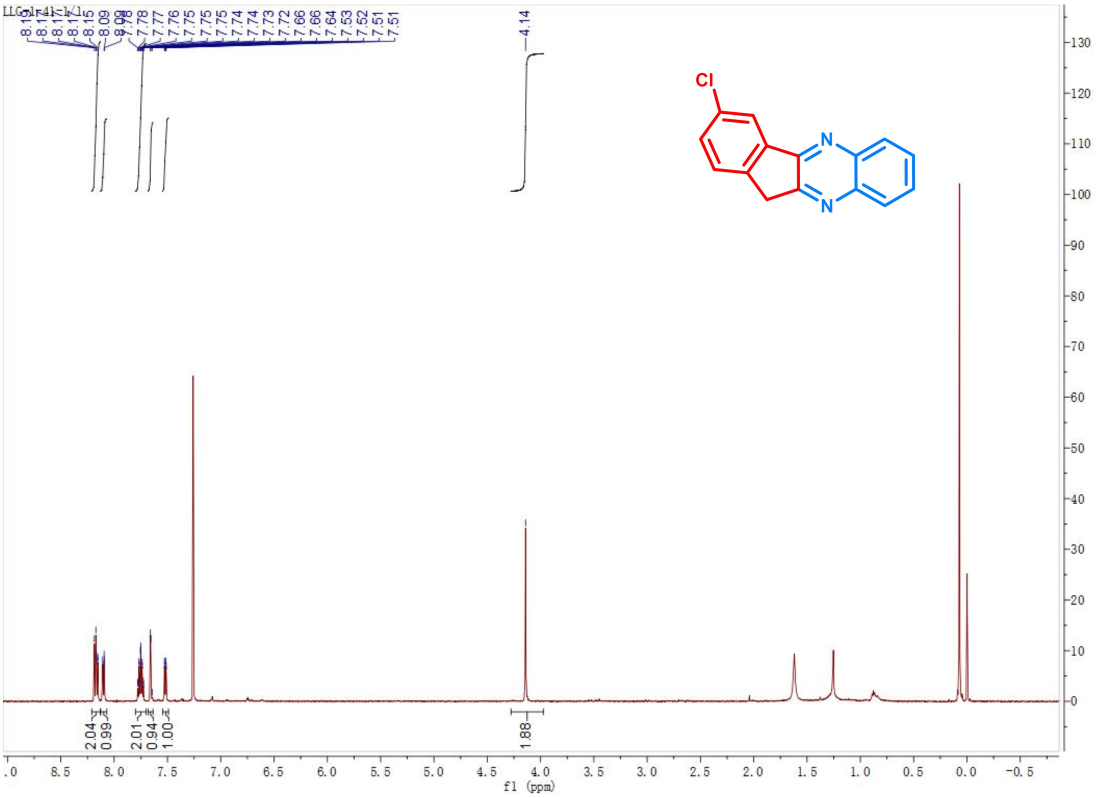

Figure S27. [1] H NMR spectrum of compound **2.3ba-1**.

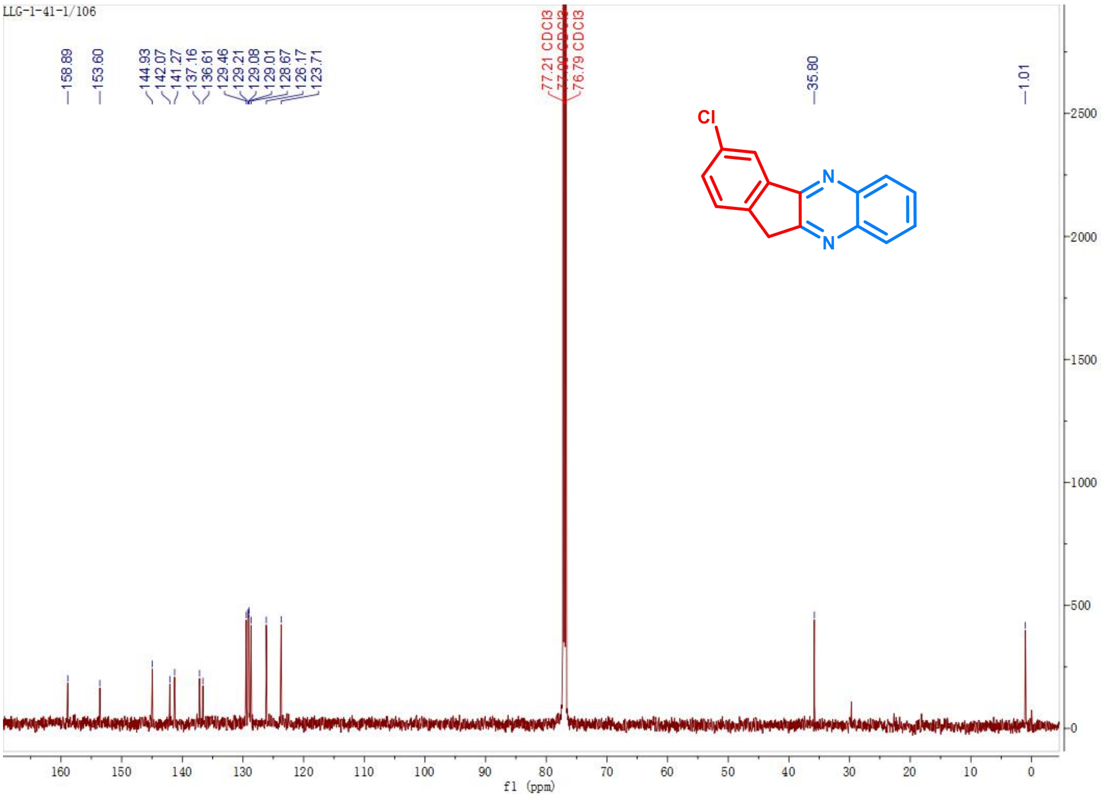

Figure S28. [13] C NMR spectrum of compound **2.3ba-1**.

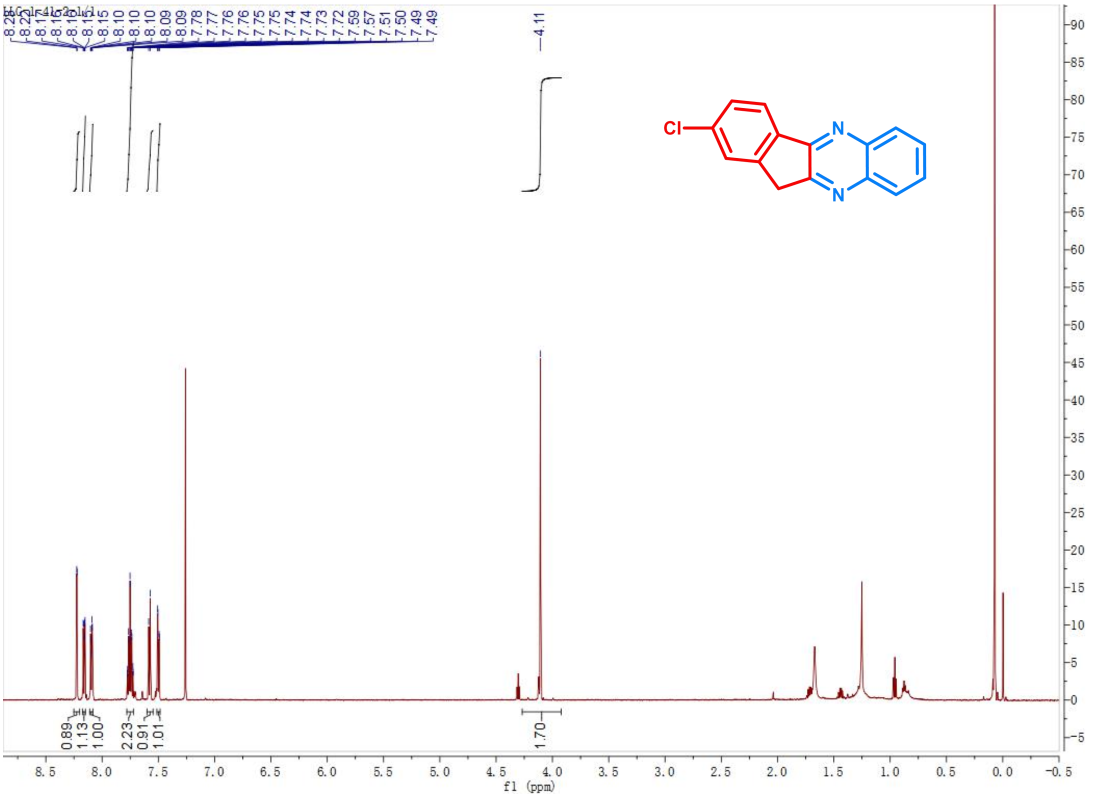

Figure S29. [1] H NMR spectrum of compound **2.3ba-2**.

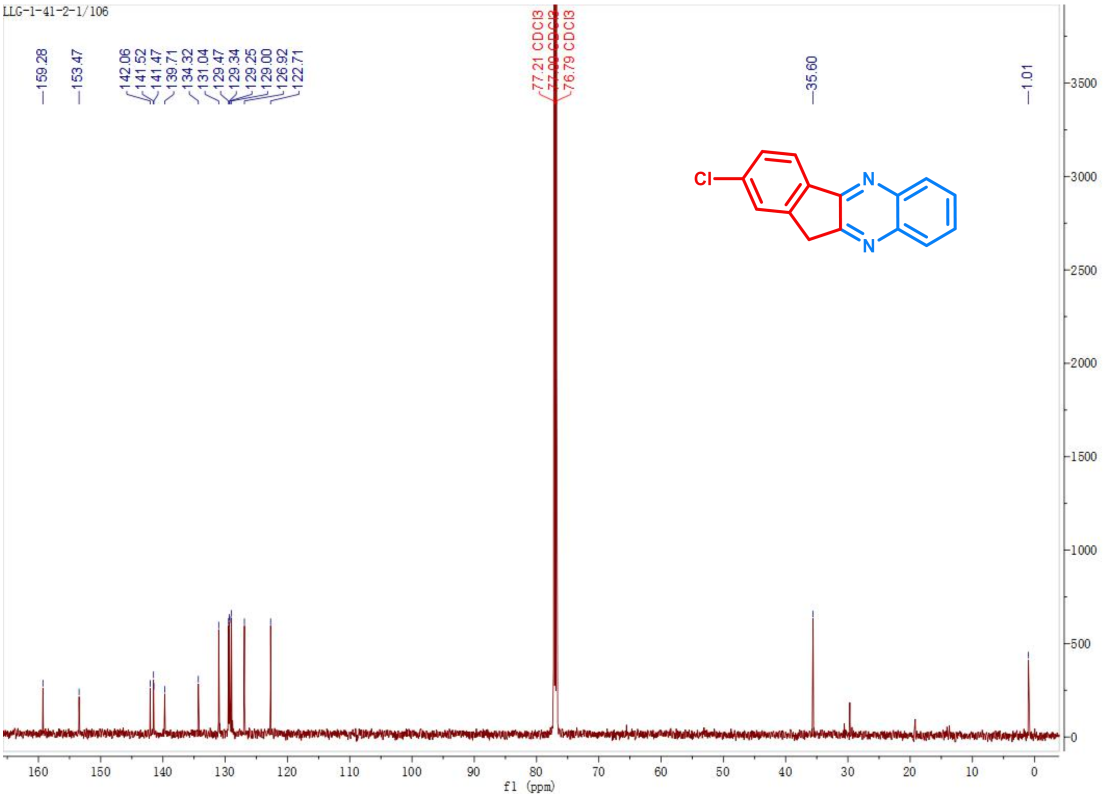

Figure S30. [13] C NMR spectrum of compound **2.3ba-2**.

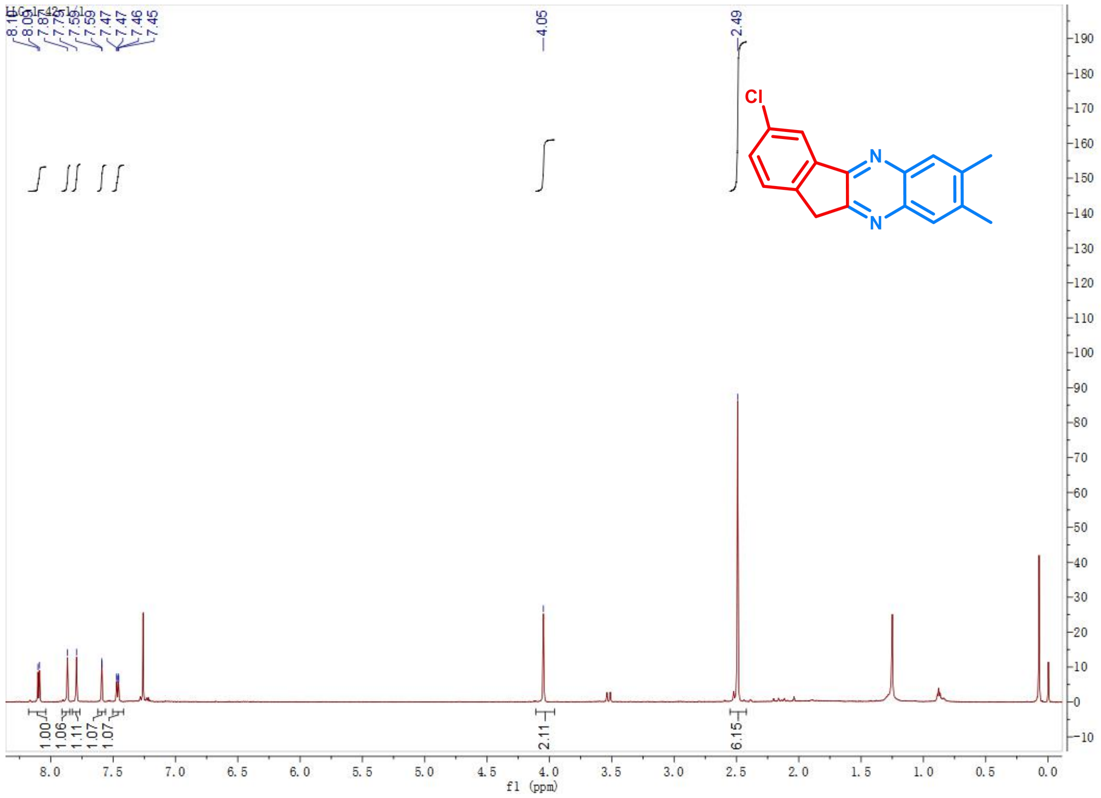

Figure S31. [1] H NMR spectrum of compound **2.3bb-1**.

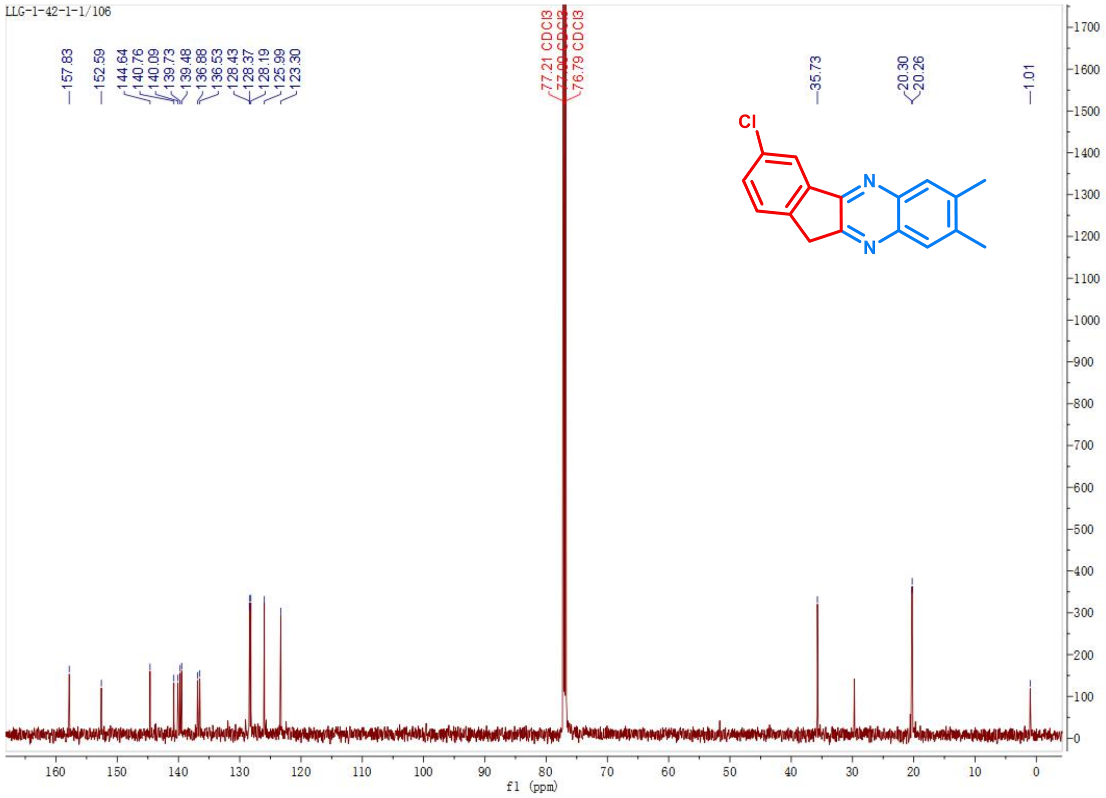

Figure S32. [13] C NMR spectrum of compound **2.3bb-1**.

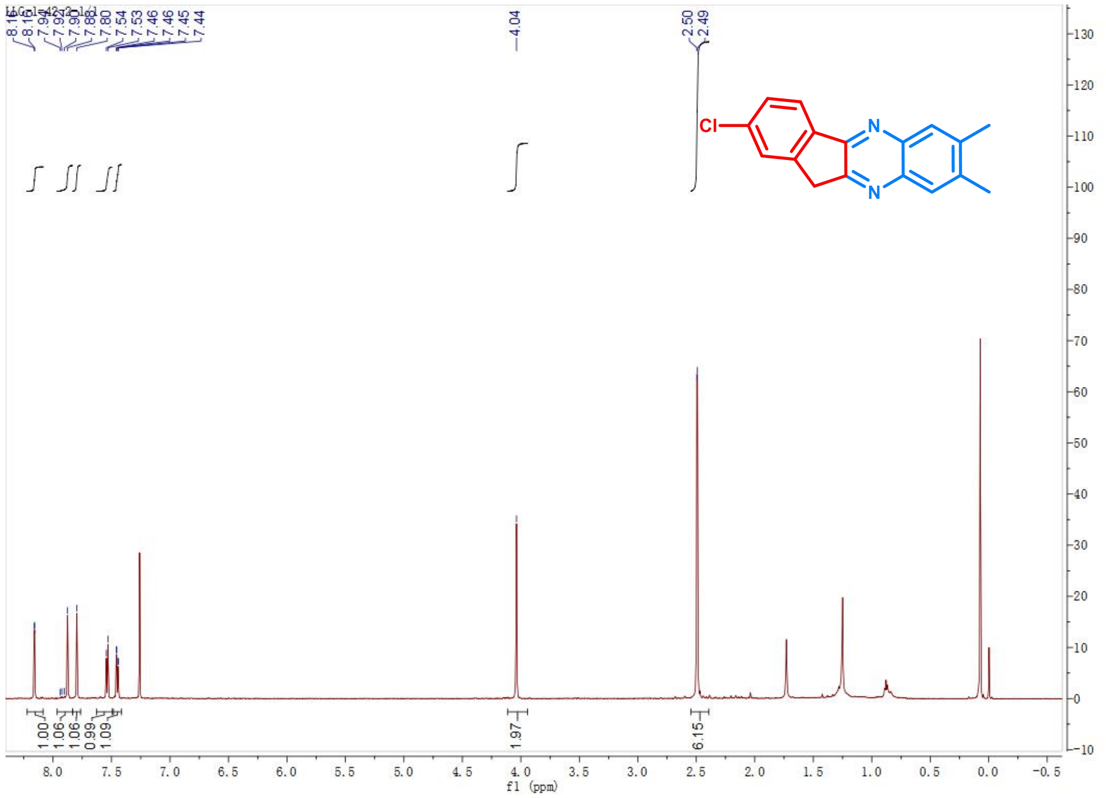

Figure S33. [1] H NMR spectrum of compound **2.3bb-2**.

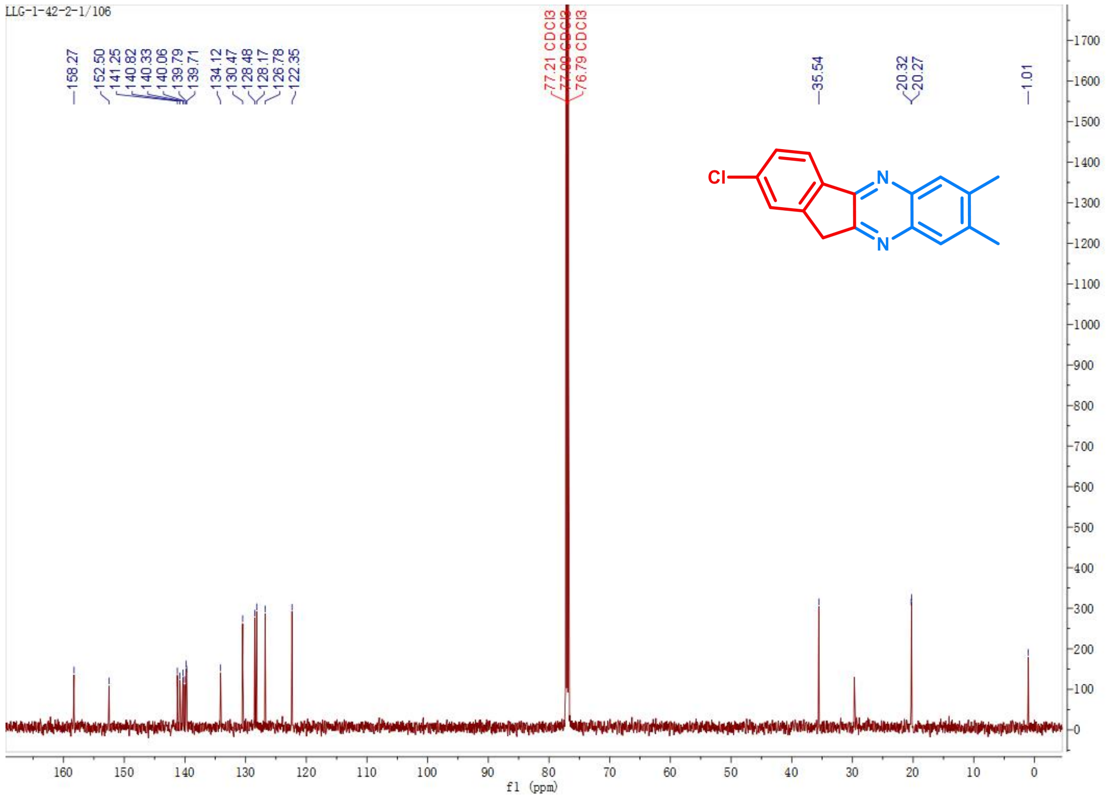

Figure S34. [13] C NMR spectrum of compound **2.3bb-2**.

Mass Spectrum SmartFormula Report

Analysis Info

Analysis Name	D:\Data\USER-2021\LLG-1-29.d	Acquisition Date	8/27/2021 2:38:12 PM
Method	tune_low_NEW.m	Operator	Ma
Sample Name	LLG-1-29	Instrument / Ser#	micrOTOF-Q II 10203
Comment			

Acquisition Parameter

Source Type	ESI	Ion Polarity	Positive	Set Nebulizer	0.8 Bar
Focus	Not active	Set Capillary	4500 V	Set Dry Heater	180 °C
Scan Begin	50 m/z	Set End Plate Offset	-500 V	Set Dry Gas	6.0 l/min
Scan End	1200 m/z	Set Collision Cell RF	150.0 Vpp	Set Divert Valve	Source

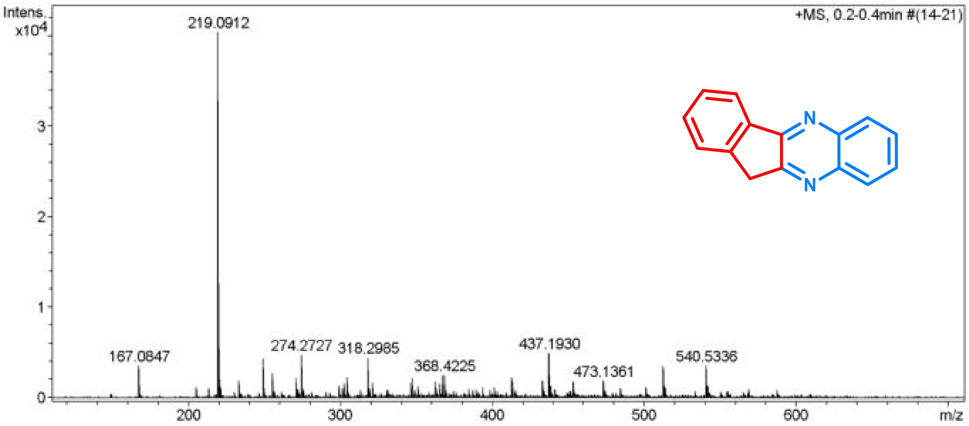

Meas. m/z	Formula	m/z	err [ppm]	mSigma	N-Rule	e⁻ Conf
219.0912						
	C 15 H 11 N 2	219.0917	2.3	9.12	ok	even

Figure S35. Mass spectrum smartFormula report of compound **2.3aa**.

Mass Spectrum SmartFormula Report

Analysis Info

Analysis Name	D:\Data\USER-2021\LLG-1-28.d	Acquisition Date	6/25/2021 2:19:37 PM
Method	tune_low_NEW.m	Operator	Ma
Sample Name	LLG-1-28	Instrument / Ser#	micrOTOF-Q II 10203
Comment			

Acquisition Parameter

Source Type	ESI	Ion Polarity	Positive	Set Nebulizer	0.8 Bar
Focus	Not active	Set Capillary	4500 V	Set Dry Heater	180 °C
Scan Begin	50 m/z	Set End Plate Offset	-500 V	Set Dry Gas	6.0 l/min
Scan End	1200 m/z	Set Collision Cell RF	150.0 Vpp	Set Divert Valve	Source

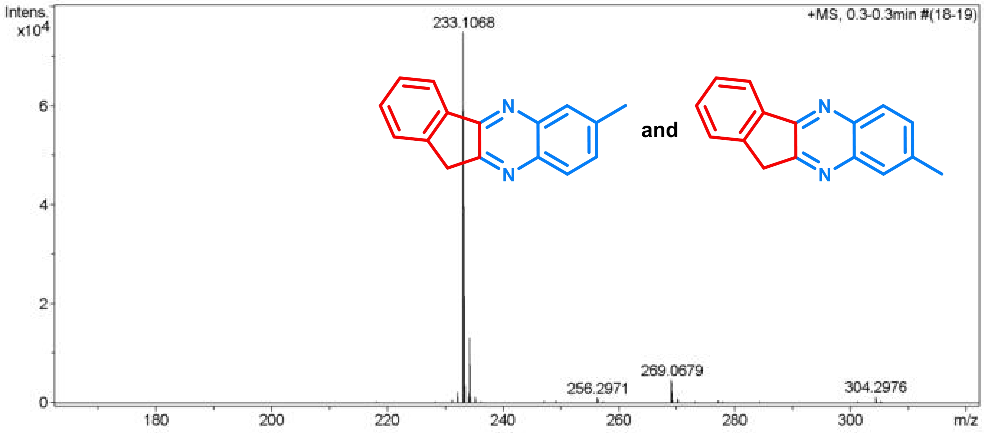

Meas. m/z	Formula	m/z	err [mDa]	err [ppm]	mSigma	N-Rule	e⁻ Conf	z
233.1068								1+
	C 16 H 13 N 2	233.1073	0.5	2.1	3.74	ok	even	

Figure S36. Mass spectrum smartFormula report of compounds **2.3ab-1** and **2.3ab-2**.

Mass Spectrum SmartFormula Report

Analysis Info

Analysis Name	D:\Data\USER-2021\LLG-1-31-1.d	Acquisition Date	6/25/2021 2:25:06 PM
Method	tune_low_NEW.m	Operator	Ma
Sample Name	LLG-1-31-1	Instrument / Ser#	micrOTOF-Q II 10203
Comment			

Acquisition Parameter

Source Type	ESI	Ion Polarity	Positive	Set Nebulizer	0.8 Bar
Focus	Not active	Set Capillary	4500 V	Set Dry Heater	180 °C
Scan Begin	50 m/z	Set End Plate Offset	-500 V	Set Dry Gas	6.0 l/min
Scan End	1200 m/z	Set Collision Cell RF	150.0 Vpp	Set Divert Valve	Source

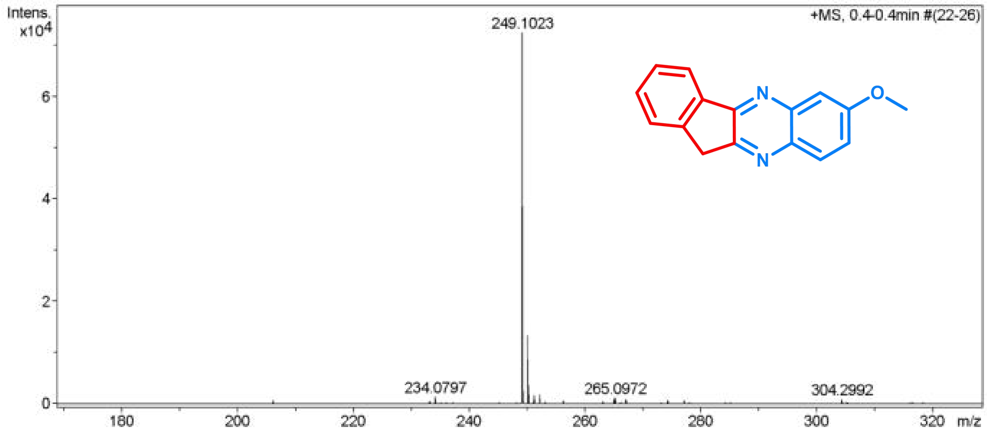

Meas. m/z	Formula	m/z	err [mDa]	err [ppm]	mSigma	N-Rule	e⁻ Conf	z
249.1023								1+
	C 16 H 13 N 2 O	249.1022	-0.0	-0.1	6.00	ok	even	

Figure S37. Mass spectrum smartFormula report of compound **2.3ac-1**.

Mass Spectrum SmartFormula Report

Analysis Info

Analysis Name	D:\Data\USER-2021\LLG-1-31-2.d	Acquisition Date	6/25/2021 2:26:59 PM
Method	tune_low_NEW.m	Operator	Ma
Sample Name	LLG-1-31-2	Instrument / Ser#	micrOTOF-Q II 10203
Comment			

Acquisition Parameter

Source Type	ESI	Ion Polarity	Positive	Set Nebulizer	0.8 Bar
Focus	Not active	Set Capillary	4500 V	Set Dry Heater	180 °C
Scan Begin	50 m/z	Set End Plate Offset	-500 V	Set Dry Gas	6.0 l/min
Scan End	1200 m/z	Set Collision Cell RF	150.0 Vpp	Set Divert Valve	Source

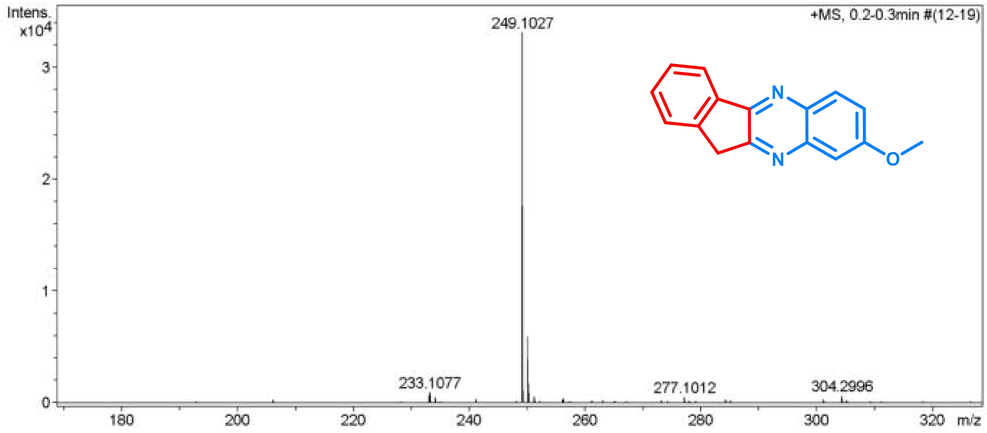

Meas. m/z	Formula	m/z	err [mDa]	err [ppm]	mSigma	N-Rule	e⁻ Conf	z
249.1027								1+
	C 16 H 13 N 2 O	249.1022	-0.4	-1.8	2.26	ok	even	

Figure S38. Mass spectrum smartFormula report of compound **2.3ac-2.**

Mass Spectrum SmartFormula Report

Analysis Info

Analysis Name	D:\data\USER-2021\LLG- 1-65-2-2.d	Acquisition Date	12/3/2021 3:12:23 PM
Method	tune_low_NEW.m	Operator	Ma
Sample Name	LLG- 1-65-2-2	Instrument / Ser#	micrOTOF-Q II 10203
Comment			

Acquisition Parameter

Source Type	ESI	Ion Polarity	Positive	Set Nebulizer	0.8 Bar
Focus	Not active	Set Capillary	4500 V	Set Dry Heater	180 °C
Scan Begin	50 m/z	Set End Plate Offset	-500 V	Set Dry Gas	6.0 l/min
Scan End	1200 m/z	Set Collision Cell RF	150.0 Vpp	Set Divert Valve	Source

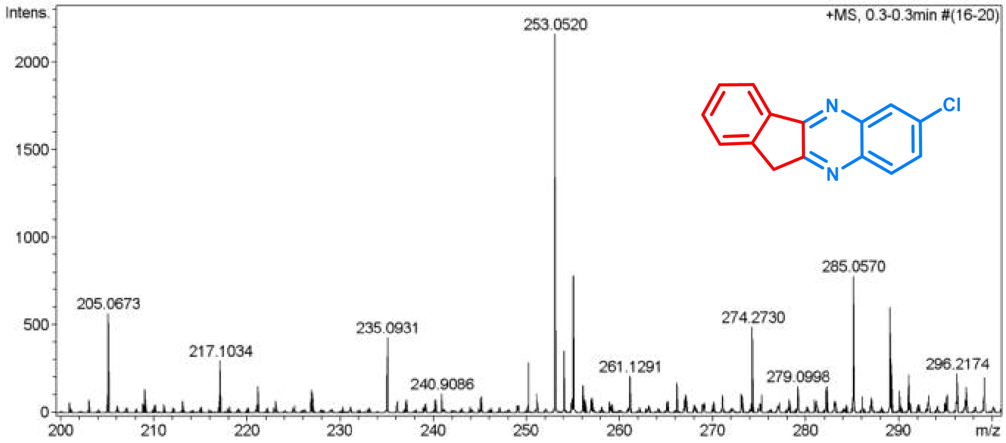

Meas. m/z	Formula	m/z	err [ppm]	mSigma	N-Rule	e⁻ Conf
253.0520						
	C 15 H 10 Cl N 2	253.0527	2.8	15.54	ok	even

Figure S39. Mass spectrum smartFormula report of compound **2.3ad-1.**

Mass Spectrum SmartFormula Report

Analysis Info

Analysis Name	D:\data\USER-2021\LLG- 1-65-1-3.d	Acquisition Date	12/3/2021 3:10:18 PM
Method	tune_low_NEW.m	Operator	Ma
Sample Name	LLG- 1-65-1-3	Instrument / Ser#	micrOTOF-Q II 10203
Comment			

Acquisition Parameter

Source Type	ESI	Ion Polarity	Positive	Set Nebulizer	0.8 Bar
Focus	Not active	Set Capillary	4500 V	Set Dry Heater	180 °C
Scan Begin	50 m/z	Set End Plate Offset	-500 V	Set Dry Gas	6.0 l/min
Scan End	1200 m/z	Set Collision Cell RF	150.0 Vpp	Set Divert Valve	Source

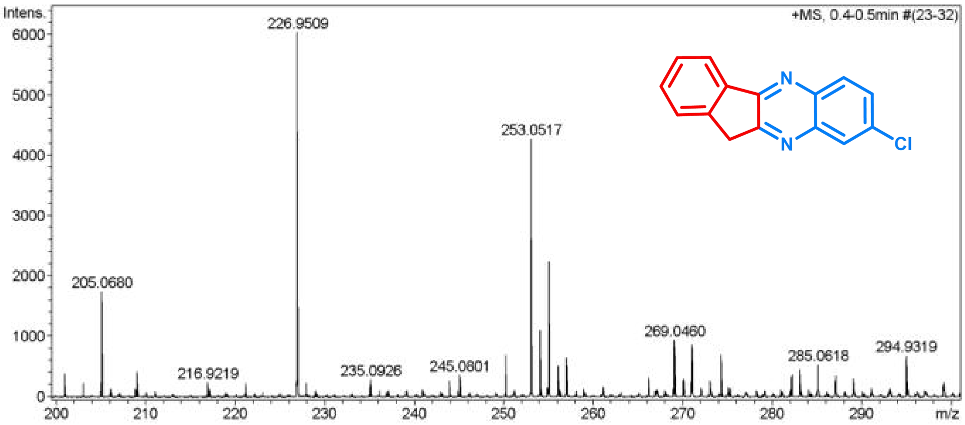

Meas. m/z	Formula	m/z	err [ppm]	mSigma	N-Rule	e⁻ Conf
253.0517						
	C 15 H 10 Cl N 2	253.0527	4.1	112.59	ok	even

Figure S40. Mass spectrum smartFormula report of compound **2.3ad-2.**

Mass Spectrum SmartFormula Report

Analysis Info

Analysis Name	D:\Data\USER-2021\LLG-1-29-2.d	Acquisition Date	6/25/2021 2:23:26 PM
Method	tune_low_NEW.m	Operator	Ma
Sample Name	LLG-1-29-2	Instrument / Ser#	micrOTOF-Q II 10203
Comment			

Acquisition Parameter

Source Type	ESI	Ion Polarity	Positive	Set Nebulizer	0.8 Bar
Focus	Not active	Set Capillary	4500 V	Set Dry Heater	180 °C
Scan Begin	50 m/z	Set End Plate Offset	-500 V	Set Dry Gas	6.0 l/min
Scan End	1200 m/z	Set Collision Cell RF	150.0 Vpp	Set Divert Valve	Source

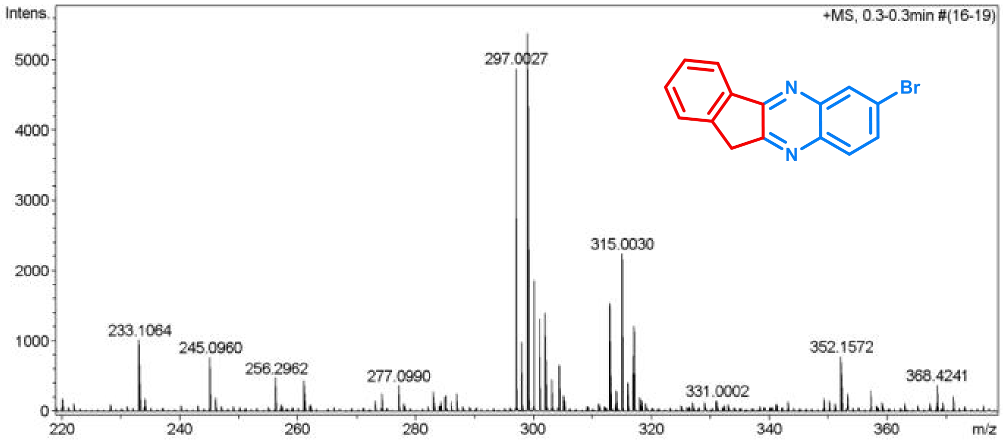

Meas. m/z	Formula	m/z	err [mDa]	err [ppm]	mSigma	N-Rule	e⁻ Conf	z
297.0027								1+
	C 15 H 10 Br N 2	297.0022	-0.5	-1.6	466.27	ok	even	

Figure S41. Mass spectrum smartFormula report of compound **2.3ae-1**.

Mass Spectrum SmartFormula Report

Analysis Info

Analysis Name	D:\Data\USER-2021\LLG-1-29-1.d	Acquisition Date	6/25/2021 2:22:01 PM
Method	tune_low_NEW.m	Operator	Ma
Sample Name	LLG-1-28	Instrument / Ser#	micrOTOF-Q II 10203
Comment			

Acquisition Parameter

Source Type	ESI	Ion Polarity	Positive	Set Nebulizer	0.8 Bar
Focus	Not active	Set Capillary	4500 V	Set Dry Heater	180 °C
Scan Begin	50 m/z	Set End Plate Offset	-500 V	Set Dry Gas	6.0 l/min
Scan End	1200 m/z	Set Collision Cell RF	150.0 Vpp	Set Divert Valve	Source

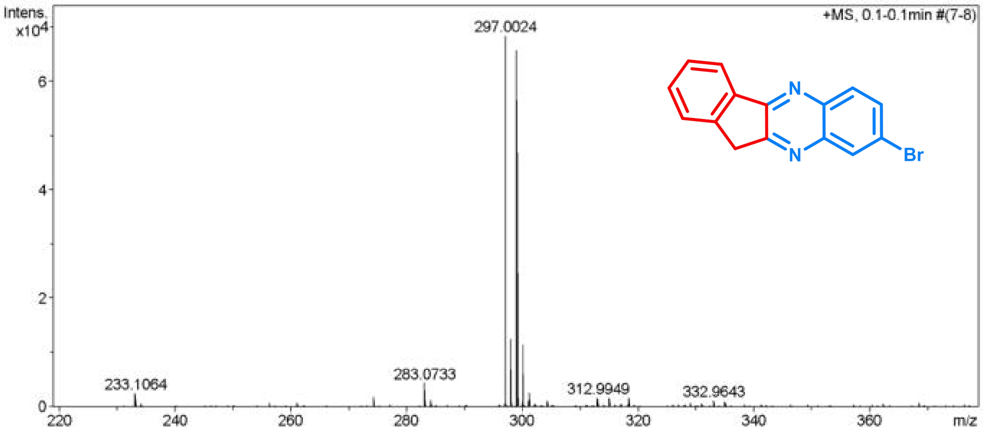

Meas. m/z	Formula	m/z	err [mDa]	err [ppm]	mSigma	N-Rule	e⁻ Conf	z
297.0024								1+
	C 15 H 10 Br N 2	297.0022	-0.2	-0.8	12.61	ok	even	

Figure S42. Mass spectrum smartFormula report of compound **2.3ae-2**.

Mass Spectrum SmartFormula Report

Analysis Info

Analysis Name	D:\Data\USER-2021\LLG-1-34.d	Acquisition Date	6/25/2021 2:28:55 PM
Method	tune_low_NEW.m	Operator	Ma
Sample Name	LLG-1-34	Instrument / Ser#	micrOTOF-Q II 10203
Comment			

Acquisition Parameter

Source Type	ESI	Ion Polarity	Positive	Set Nebulizer	0.8 Bar
Focus	Not active	Set Capillary	4500 V	Set Dry Heater	180 °C
Scan Begin	50 m/z	Set End Plate Offset	-500 V	Set Dry Gas	6.0 l/min
Scan End	1200 m/z	Set Collision Cell RF	150.0 Vpp	Set Divert Valve	Source

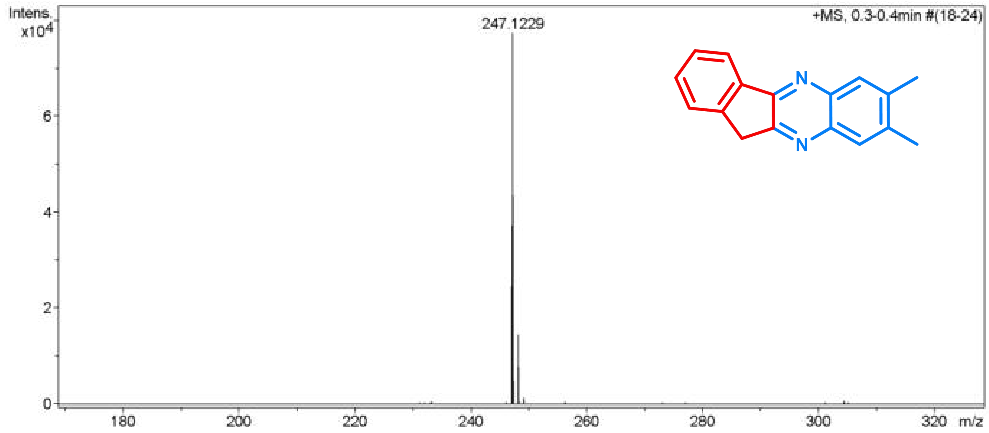

Meas. m/z	Formula	m/z	err [mDa]	err [ppm]	mSigma	N-Rule	e⁻ Conf	z
247.1229								1+
	C 17 H 15 N 2	247.1230	0.1	0.5	2.28	ok	even	

Figure S43. Mass spectrum smartFormula report of compound **2.3af.**

Mass Spectrum SmartFormula Report

Analysis Info

Analysis Name	D:\Data\USER-2021\LLG-1-32.d	Acquisition Date	6/25/2021 2:31:15 PM
Method	tune_low_NEW.m	Operator	Ma
Sample Name	LLG-1-32	Instrument / Ser#	micrOTOF-Q II 10203
Comment			

Acquisition Parameter

Source Type	ESI	Ion Polarity	Positive	Set Nebulizer	0.8 Bar
Focus	Not active	Set Capillary	4500 V	Set Dry Heater	180 °C
Scan Begin	50 m/z	Set End Plate Offset	-500 V	Set Dry Gas	6.0 l/min
Scan End	1200 m/z	Set Collision Cell RF	150.0 Vpp	Set Divert Valve	Source

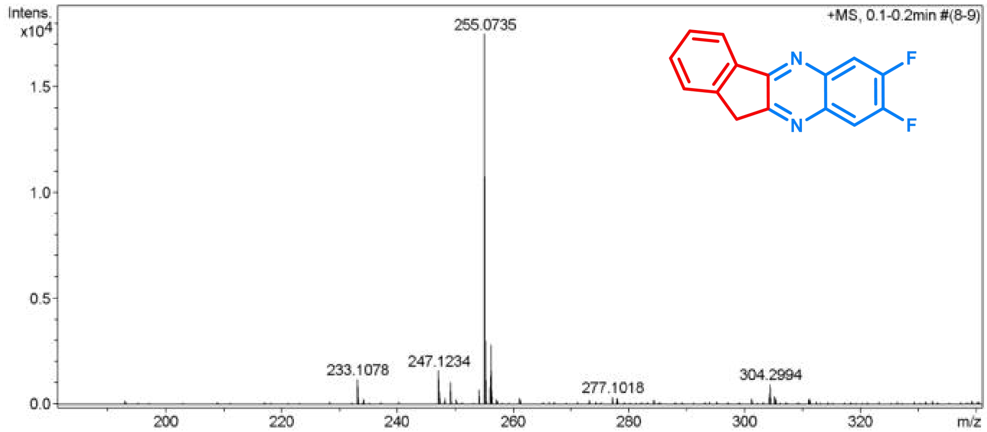

Meas. m/z	Formula	m/z	err [mDa]	err [ppm]	mSigma	N-Rule	e⁻ Conf	z
255.0735								1+
	C 15 H 9 F 2 N 2	255.0728	-0.7	-2.6	5.31	ok	even	

Figure S44. Mass spectrum smartFormula report of compound **2.3ag.**

Mass Spectrum SmartFormula Report

Analysis Info

Analysis Name	D:\Data\USER-2021\LLG-1-39.d	Acquisition Date	6/25/2021 2:32:33 PM
Method	tune_low_NEW.m	Operator	Ma
Sample Name	LLG-1-39	Instrument / Ser#	micrOTOF-Q II 10203
Comment			

Acquisition Parameter

Source Type	ESI	Ion Polarity	Positive	Set Nebulizer	0.8 Bar
Focus	Not active	Set Capillary	4500 V	Set Dry Heater	180 °C
Scan Begin	50 m/z	Set End Plate Offset	-500 V	Set Dry Gas	6.0 l/min
Scan End	1200 m/z	Set Collision Cell RF	150.0 Vpp	Set Divert Valve	Source

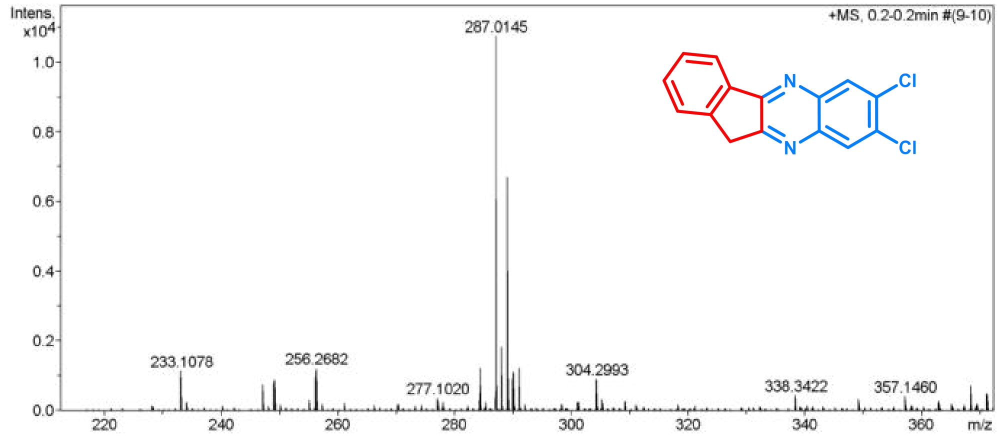

Meas. m/z	Formula	m/z	err [mDa]	err [ppm]	mSigma	N-Rule	e⁻ Conf	z
287.0145								1+
	C 15 H 9 Cl 2 N 2	287.0137	-0.7	-2.6	12.40	ok	even	

Figure S45. Mass spectrum smartFormula report of compound **2.3ah.**

Mass Spectrum SmartFormula Report

Analysis Info

Analysis Name	D:\Data\USER-2021\LLG-1-40.d	Acquisition Date	6/25/2021 2:34:34 PM
Method	tune_low_NEW.m	Operator	Ma
Sample Name	LLG-1-40	Instrument / Ser#	micrOTOF-Q II 10203
Comment			

Acquisition Parameter

Source Type	ESI	Ion Polarity	Positive	Set Nebulizer	0.8 Bar
Focus	Not active	Set Capillary	4500 V	Set Dry Heater	180 °C
Scan Begin	50 m/z	Set End Plate Offset	-500 V	Set Dry Gas	6.0 l/min
Scan End	1200 m/z	Set Collision Cell RF	150.0 Vpp	Set Divert Valve	Source

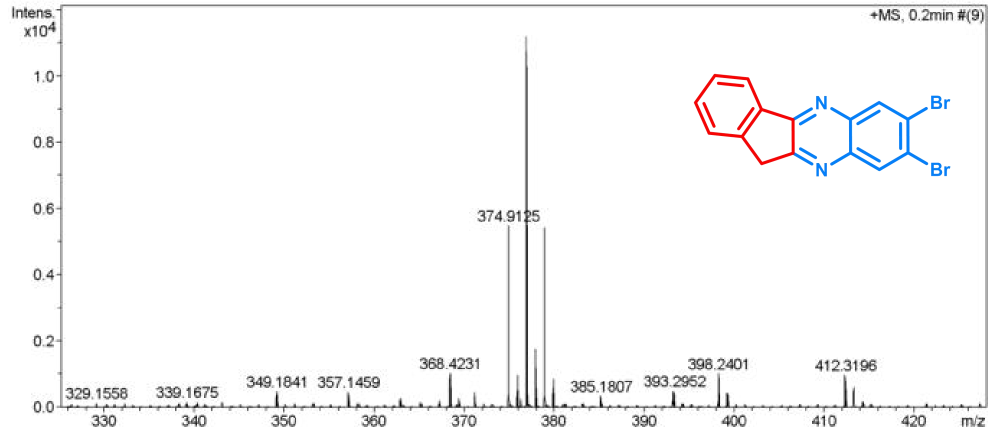

Meas. m/z	Formula	m/z	err [mDa]	err [ppm]	mSigma	N-Rule	e⁻ Conf	z
374.9125								1+
	C 15 H 9 Br 2 N 2	374.9127	0.2	0.6	466.03	ok	even	

Figure S46. Mass spectrum smartFormula report of compound **2.3ai.**

Mass Spectrum SmartFormula Report

Analysis Info

Analysis Name	D:\Data\USER-2021\LLG-1-33.d	Acquisition Date	6/25/2021 2:36:00 PM
Method	tune_low_NEW.m	Operator	Ma
Sample Name	LLG-1-33	Instrument / Ser#	micrOTOF-Q II 10203
Comment			

Acquisition Parameter

Source Type	ESI	Ion Polarity	Positive	Set Nebulizer	0.8 Bar
Focus	Not active	Set Capillary	4500 V	Set Dry Heater	180 °C
Scan Begin	50 m/z	Set End Plate Offset	-500 V	Set Dry Gas	6.0 l/min
Scan End	1200 m/z	Set Collision Cell RF	150.0 Vpp	Set Divert Valve	Source

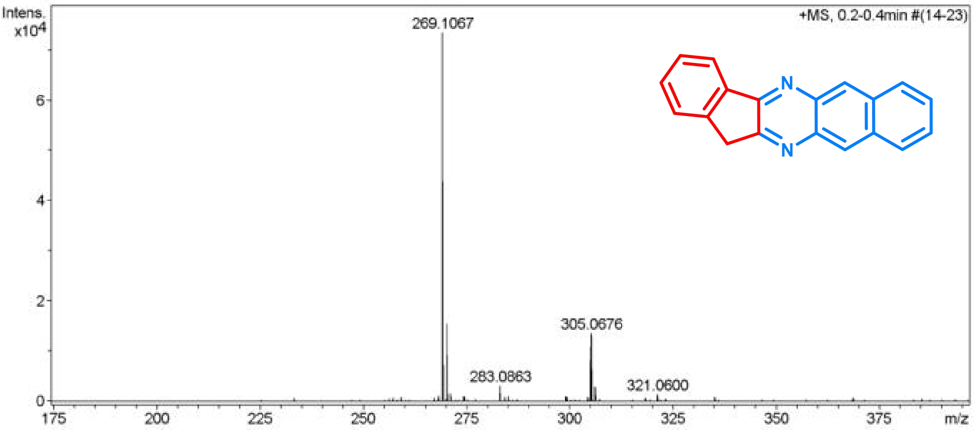

Meas. m/z	Formula	m/z	err [mDa]	err [ppm]	mSigma	N-Rule	e¯ Conf	z
269.1067								1+
	C 19 H 13 N 2	269.1073	0.6	2.2	1.29	ok	even	

Figure S47. Mass spectrum smartFormula report of compound **2.3aj.**

Mass Spectrum SmartFormula Report

Analysis Info

Analysis Name	D:\data\USER-2021\LLG-1-41-1.d	Acquisition Date	6/25/2021 2:39:43 PM
Method	tune_low_NEW.m	Operator	Ma
Sample Name	LLG-1-41-1	Instrument / Ser#	micrOTOF-Q II 10203
Comment			

Acquisition Parameter

Source Type	ESI	Ion Polarity	Positive	Set Nebulizer	0.8 Bar
Focus	Not active	Set Capillary	4500 V	Set Dry Heater	180 °C
Scan Begin	50 m/z	Set End Plate Offset	-500 V	Set Dry Gas	6.0 l/min
Scan End	1200 m/z	Set Collision Cell RF	150.0 Vpp	Set Divert Valve	Source

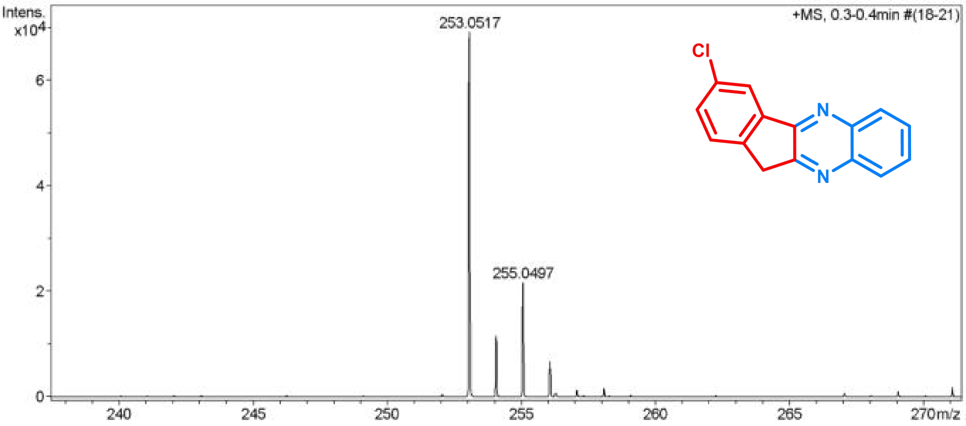

Meas. m/z	Formula	m/z	err [ppm]	mSigma	N-Rule	e¯ Conf
253.0517						
	C 15 H 10 Cl N 2	253.0527	3.8	21.46	ok	even

Figure S48. Mass spectrum smartFormula report of compound **2.3ba-1.**

Mass Spectrum SmartFormula Report

Analysis Info

Analysis Name	D:\Data\USER-2021\LLG-1-41-2.d	Acquisition Date	6/25/2021 2:41:38 PM
Method	tune_low_NEW.m	Operator	Ma
Sample Name	LLG-1-41-2	Instrument / Ser#	micrOTOF-Q II 10203
Comment			

Acquisition Parameter

Source Type	ESI	Ion Polarity	Positive	Set Nebulizer	0.8 Bar
Focus	Not active	Set Capillary	4500 V	Set Dry Heater	180 °C
Scan Begin	50 m/z	Set End Plate Offset	-500 V	Set Dry Gas	6.0 l/min
Scan End	1200 m/z	Set Collision Cell RF	150.0 Vpp	Set Divert Valve	Source

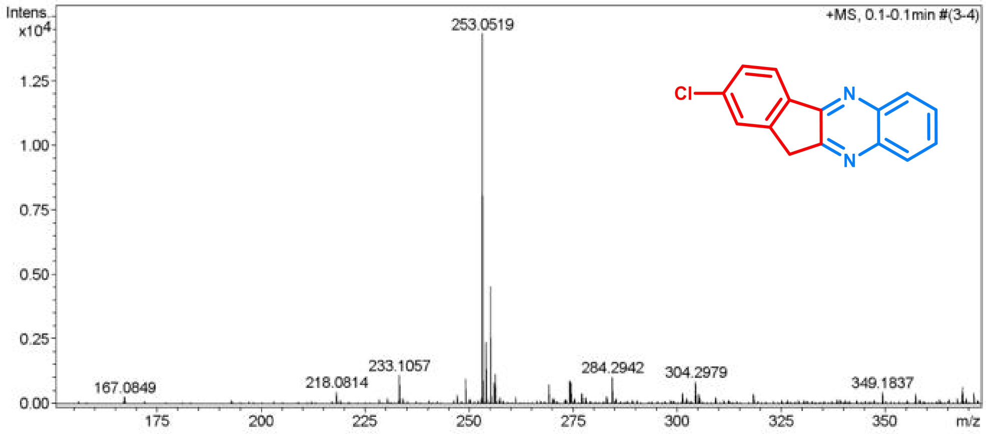

Meas. m/z	Formula	m/z	err [mDa]	err [ppm]	mSigma	N-Rule	e⁻ Conf	z
253.0519								1+
	C 15 H 10 Cl N 2	253.0527	0.8	3.3	7.19	ok	even	

Figure S49. Mass spectrum smartFormula report of compound **2.3ba-2.**

Mass Spectrum SmartFormula Report

Analysis Info

Analysis Name	D:\Data\USER-2021\LLG-1-42-2.d	Acquisition Date	6/25/2021 2:44:04 PM
Method	tune_low_NEW.m	Operator	Ma
Sample Name	LLG-1-42-2	Instrument / Ser#	micrOTOF-Q II 10203
Comment			

Acquisition Parameter

Source Type	ESI	Ion Polarity	Positive	Set Nebulizer	0.8 Bar
Focus	Not active	Set Capillary	4500 V	Set Dry Heater	180 °C
Scan Begin	50 m/z	Set End Plate Offset	-500 V	Set Dry Gas	6.0 l/min
Scan End	1200 m/z	Set Collision Cell RF	150.0 Vpp	Set Divert Valve	Source

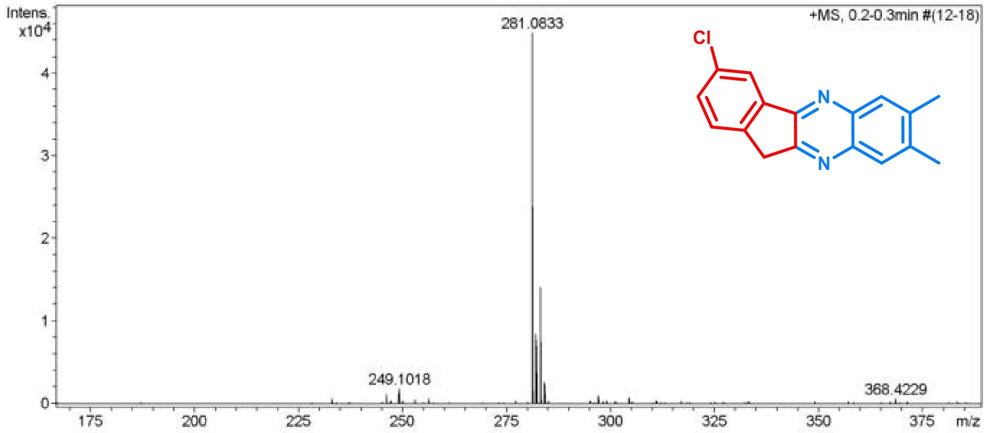

Meas. m/z	Formula	m/z	err [mDa]	err [ppm]	mSigma	N-Rule	e⁻ Conf	z
281.0833								1+
	C 17 H 14 Cl N 2	281.0840	0.7	2.4	10.12	ok	even	

Figure S50. Mass spectrum smartFormula report of compound **2.3bb-1.**

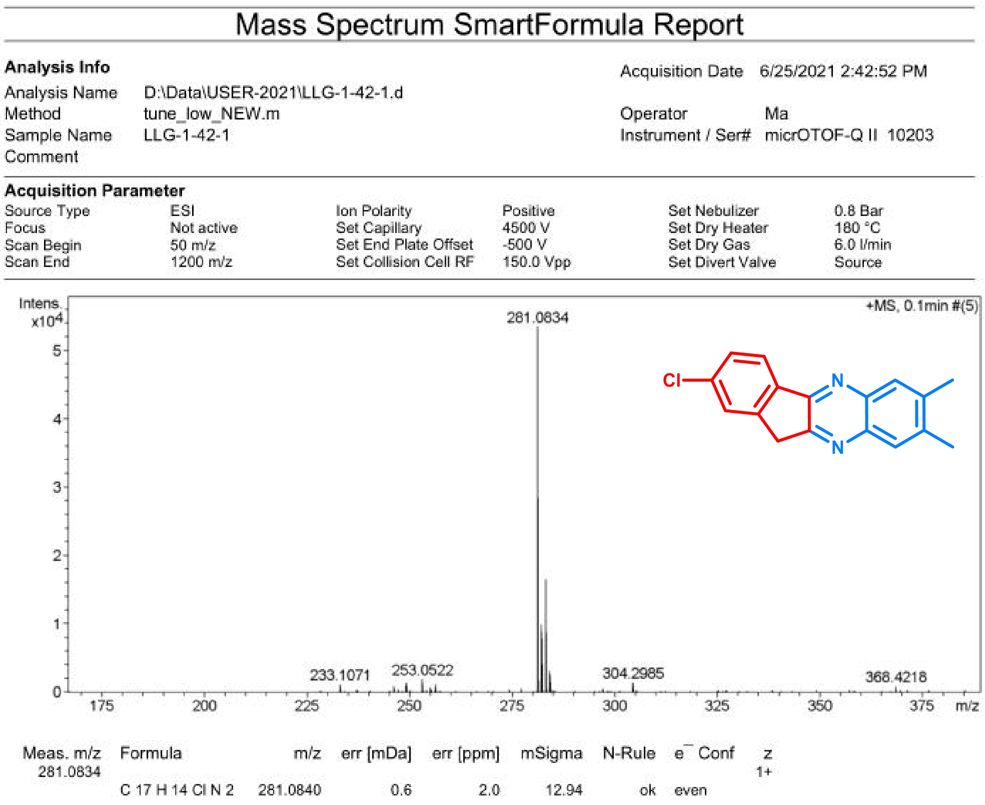

Figure S51. Mass spectrum smartFormula report of compound **2.3bb-2.**

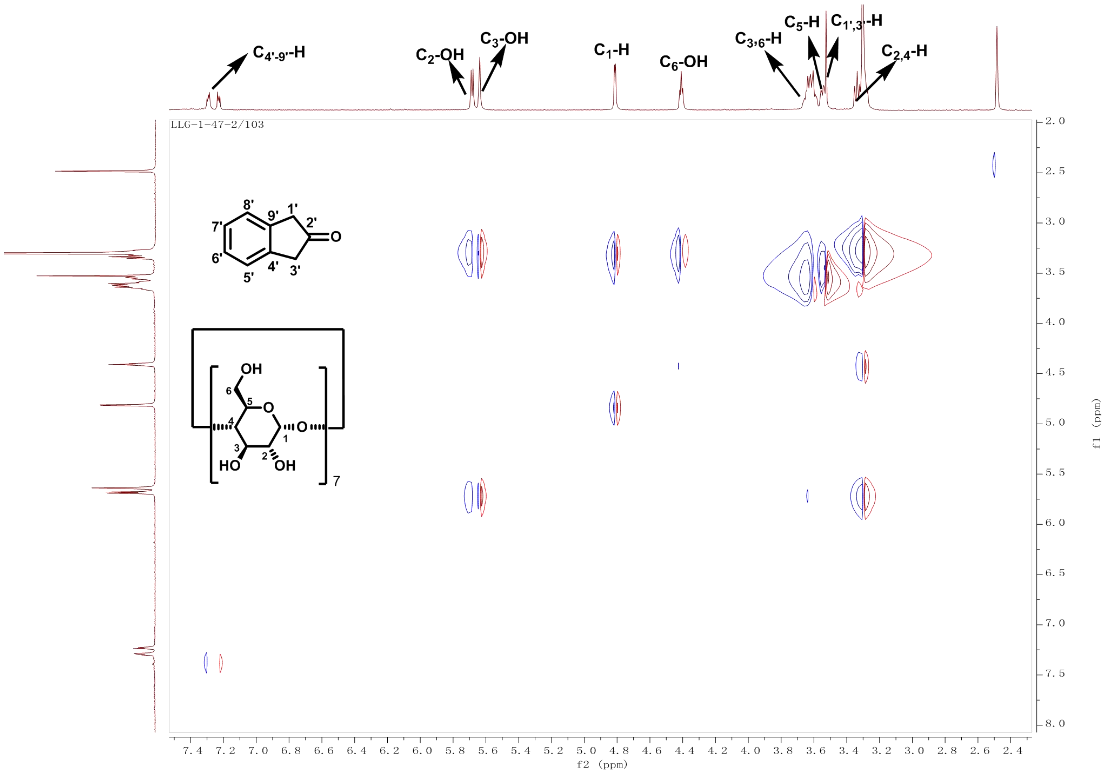

Figure S52. The NOESY spectrum of 2-indenone (1.0 eqv.) and β-CD (15 mol%) mixed after stirring in water for 12h.

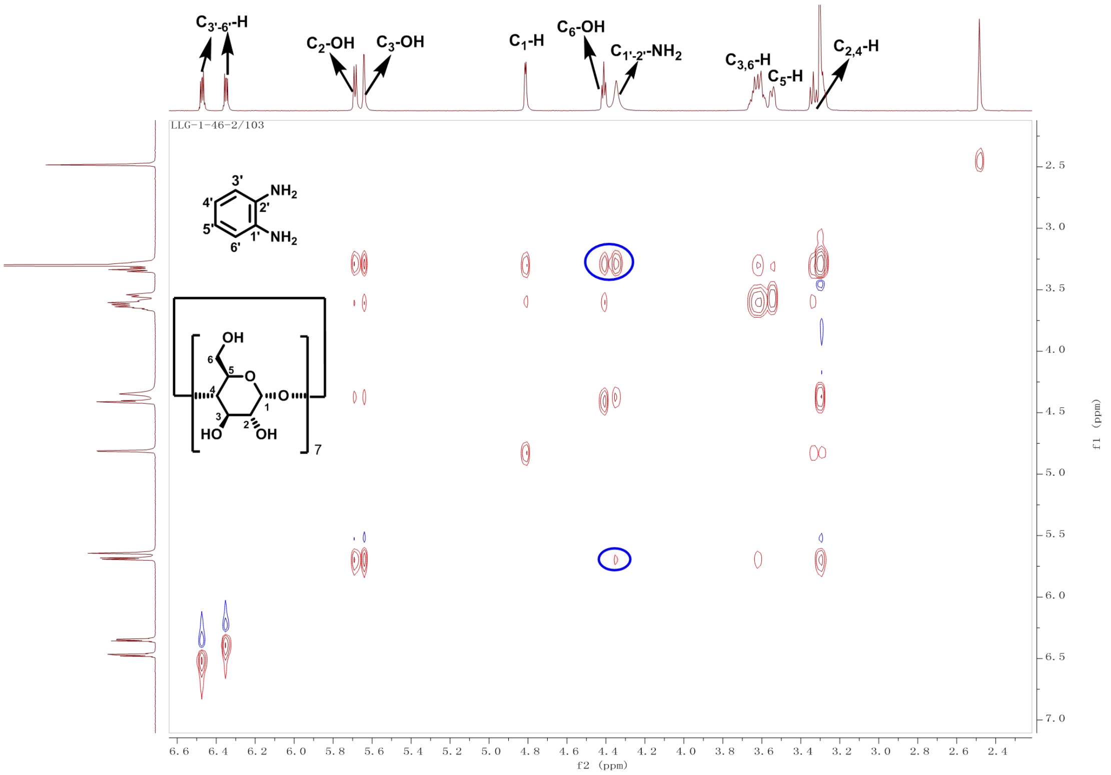

Figure S53. The NOESY spectrum of OPD (1.2 eqv.) and *β*-CD (15 mol%) mixed after stirring in water for 12h.

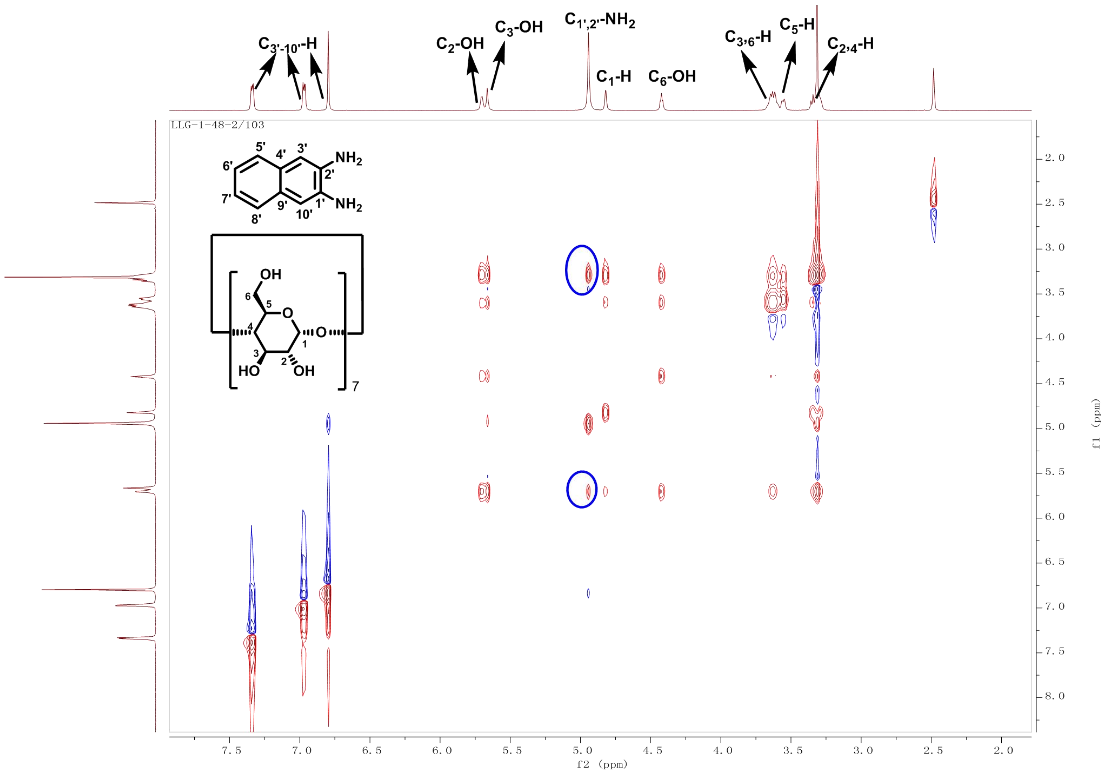

Figure S54. The NOESY spectrum of 2,3-diaminonaphthalene (1.2 eqv.) and *β*-CD (15 mol%) mixed after stirring in water for 12h.

Created by: Waters, Waters

Created on: Jan 04, 2022

Item name: MS-20220104

Created time: 14:28:42 China Standard Time

Analysis Information

Item name:	MS-20220104	Analysis Method Item name:	MS-NOPDA-0916
Version:	3	Analysis Method Version:	2
Modified date:	Jan 04, 2022 14:25:44 China Standard Time	Sample Set Created date:	Jan 04, 2022 11:07:11 China Standard Time
Modified by:	Waters, Waters	Sample Set Instrument system name:	UPLC_QTof_2
Folder:	Company/sample/2022		

MS Instrument Type: Waters Vion® IMS QTof
Experiment Settings:

Experiment type:	ESI+	Scan Mode:MS	
Capillary voltage:	3.0 kv	Low mass:50m/z	
Soure temperature:	120°C	High mass:2000m/z	
Desolvation temperature:	450°C	Scan time:0.200s	
Cone gas:	50L/h		
Desolvation gas:	800L/h		

Item name: 20220104-LLG20220103, Sample position: 1:A,8, Replicate number: 1

.	Formula	Neutral mass (Da)	Observed m/z	Mass error (mDa)	Mass error (ppm)	Response	Adducts	Identification status
1	C15H14N2	222.11570	223.12199	-1.0	-4.4	67393	+H	Identified
2	C15H12N2O	236.09496	237.10165	-0.6	-2.5	3955403	+H, -e	Identified

Item name: 20220104-LLG20220103
Item description:

Channel name: Time 0.1835 +/- 0.0500 minutes

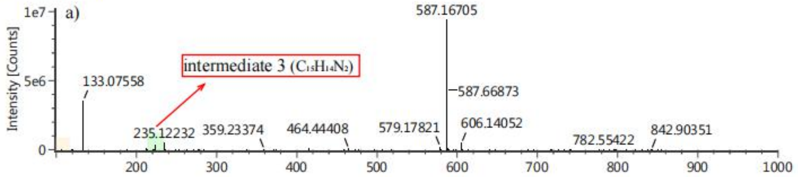

Item name: 20220104-LLG20220103
Item description:

Channel name: Time 0.1233 +/- 0.0500 minutes

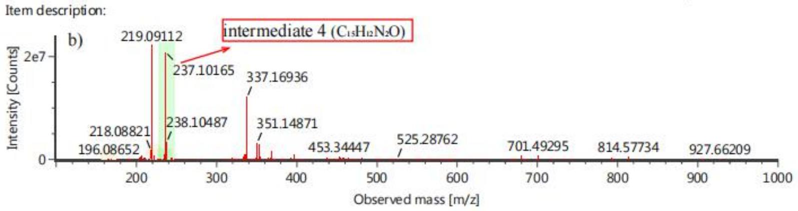

Item name: 20220104-LLG20220103
Item description:

Channel name: Time 0.1835 +/- 0.0500 minutes

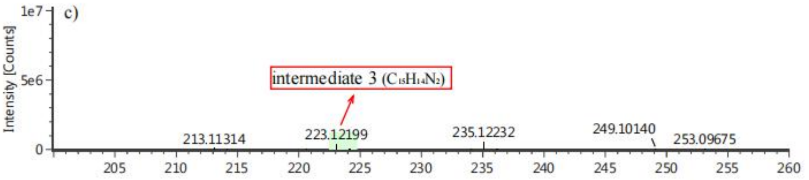

Item name: 20220104-LLG20220103
Item description:

Channel name: Time 0.1233 +/- 0.0500 minutes

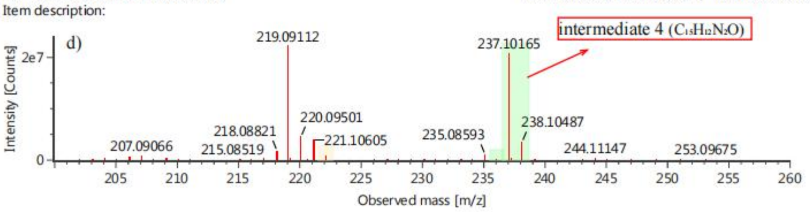

Figure S55. The HRMS of template reaction (**2.1a** (0.2 mmol), **2.2a** (0.24 mmol) and β-CD (15 %) in air) in water after 1h: **a)** the extraction mass spectrometry of intermediate **3**; **b)** the extraction mass spectrometry of intermediate **4**; **c)** the enlarged view of the abscissa of **Figure S55a**; **c)** the enlarged view of the abscissa of **Figure S55b**.
